# New hPSC SOX9 and INS Reporter Cell Lines Facilitate the Observation and Optimization of Differentiation into Insulin-Producing Cells

**DOI:** 10.1007/s12015-021-10232-9

**Published:** 2021-08-19

**Authors:** Rabea Dettmer, Isabell Niwolik, Ilir Mehmeti, Anne Jörns, Ortwin Naujok

**Affiliations:** Institute of Clinical Biochemistry, Hannover Medical School, 30625 Hannover, Germany

**Keywords:** Human pluripotent stem cells, Reporter cells, SOX9, Insulin, Stem cell-derived beta cells

## Abstract

Differentiation of human pluripotent stem cells into insulin-producing stem cell-derived beta cells harbors great potential for research and therapy of diabetes. *SOX9* plays a crucial role during development of the pancreas and particularly in the development of insulin-producing cells as SOX9^+^ cells form the source for NEUROG3^+^ endocrine progenitor cells. For the purpose of easy monitoring of differentiation efficiencies into pancreatic progenitors and insulin-producing cells, we generated new reporter lines by knocking in a P2A-H-2K^k^-F2A-GFP2 reporter gene into the *SOX9*-locus and a P2A-mCherry reporter gene into the *INS*-locus mediated by CRISPR/CAS9-technology. The knock-ins enabled co-expression of the endogenous and reporter genes and report on the endogenous gene expression. Furthermore, FACS and MACS enabled the purification of pancreatic progenitors and insulin-producing cells. Using these cell lines, we established a new differentiation protocol geared towards SOX9^+^ cells to efficiently drive human pluripotent stem cells into glucose-responsive beta cells. Our new protocol offers an alternative route towards stem cell-derived beta cells, pointing out the importance of Wnt/beta-catenin inhibition and the efficacy of EGF for the development of pancreatic progenitors, as well as the significance of 3D culture for the functionality of the generated beta cells.

**Graphic Abstract:**

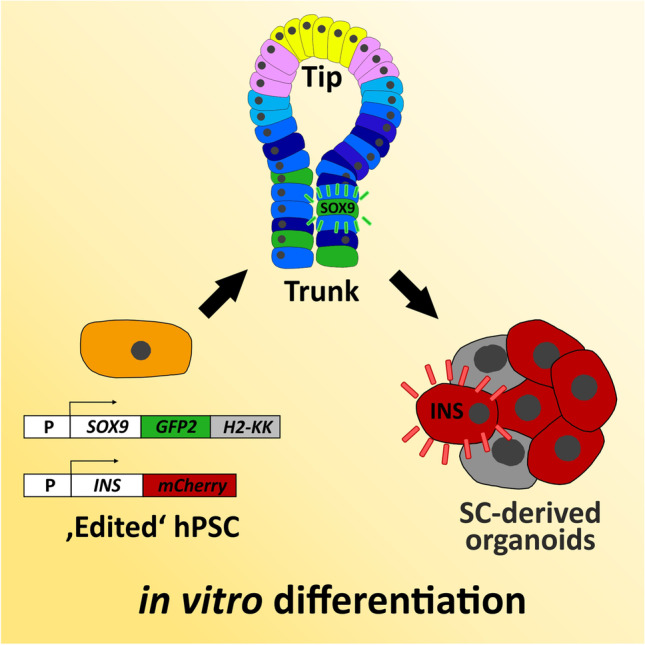

**Supplementary Information:**

The online version contains supplementary material available at 10.1007/s12015-021-10232-9.

## Introduction

The SOX9 protein belongs to a family of high-mobility domain transcription factors with pleiotropic functions during development, cellular maintenance and disease development [1]. In humans, SOX9 haploinsufficiency leads to campomelic dysplasia with pancreatic dysmorphogenesis [2]. These and other results have shown that SOX9 maintains the multipotent pancreatic progenitor pool and belongs to the group of master regulators of pancreatic development [3–6].

SOX9 expression during the initial forming of the dorsal and ventral pancreatic buds co-localizes with PDX1 in mouse and man [6, 7]*.* In mice, when the early unpolarized epithelium branches into a plexus with proximal trunk and distal tip domains, Sox9 is co-expressed with Cpa1 and Pdx1 in the tip domain or with Nkx6.1 and Pdx1 in the trunk domain [6]. The distal tip domain is considered as the predominant cellular pool for acinar differentiation, whereas the proximal trunk domain harbors the development niche for bipotent ductal/endocrine precursor cells [8]. Bipotent precursors then give rise to Neurog3^+^ cells, which are the progenitor population of all endocrine islet cell types including insulin-producing beta cells. Later, during mid second transition, Sox9 expression recedes from the tip domain and becomes restricted to the proximal trunk cells. By late gestation and in adults, Sox9 expression is confined to centroacinar and ductal cells [1, 6].

Thus, SOX9 is an interesting target for the generation of new human pluripotent stem cell (hPSC) reporter lines in order to target the development niche that gives rise to the endocrine lineage. This would open up new opportunities to develop efficient differentiation protocols for the generation of stem cell-derived beta cells or organoids, respectively (SC-derived beta cells/SC-derived organoids). Therefore, the aims of this study were to generate two types of reporter cell lines, namely a *SOX9* and a *SOX9*/*INS* knock-in cell line and a differentiation protocol optimized towards the generation of SOX9^+^ multipotent pancreatic progenitor cells (MPCs).

Insulin in the body is exclusively expressed in beta cells. The *INS* gene on chromosom 11 contains three exons and two introns [9], which encode the signal peptide, the B-chain, C-peptide, and the A-chain of the protein. *INS* expression generates biologically active insulin and C-peptide after proteolytic cleavage of the preproinsulin precursor. To introduce reporter genes into the aforementioned loci, we used CRISPR/Cas9 to generate DNA double-strand breaks around the stop codons of each gene. Then we utilized the HDR DNA-damage repair system of the cell to knock-in reporter genes. GFP2 and the surface antigen H-2K^k^ were introduced into the *SOX9*-locus and mCherry into the *INS*-locus. This permitted monitoring and cell purification of *SOX9* and *INS* expressing populations using a combined 2D/3D differentiation protocol. We can show that PDX1^+^ pancreatic-duodenal cells [10, 11], effectively differentiate into SOX9^+^ MPCs with co-expression of CPA1 or NKX6.1. By use of our triple knock-in cell line we could then track the conversion of SOX9 MPCs into SC-derived beta cells.

## Materials and Methods

### Cell Culture

The hPSC lines Hes-3 (‘ESC’) from ES Cell International and Phoenix (‘iPSC’, MHHi001-A) [12] were cultured on cell culture plastic coated with Matrigel (Corning, Amsterdam, Netherlands) and mTeSR1 (Stem Cell Technologies, Cologne, Germany) or StemMACS™ iPS-Brew XF medium (Miltenyi Biotec, Bergisch Gladbach, Germany) in an incubator with 37 °C and 5% CO_2_. Passaging was performed once a week in clusters 1:20 up to 1:40. EndoC-βH1 cells were cultured according to a standard protocol [13]. HES-3 cell lines: Organism: Homo sapiens, Cell line: RRID: CVCL_7158; Phoenix cell line: Organism: Homo sapiens, Cell line: RRID:CVCL_QX51.

### Generation of Reporter Cells

**Fig. 1:**
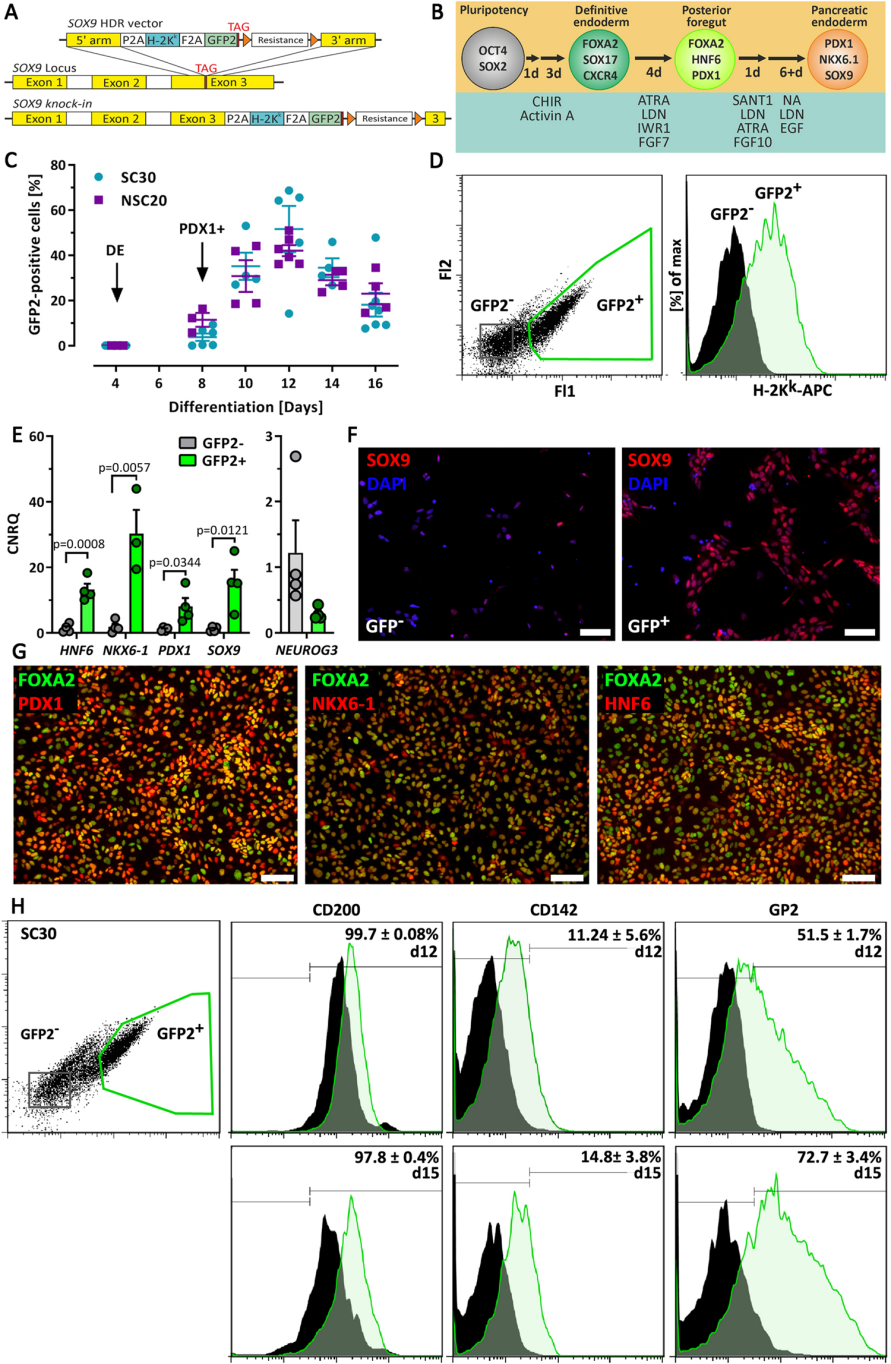
**A** Schematic presentation of the SOX9 HDR vector and the human *SOX9*-locus before and after homologous recombination. **B** Schematic presentation of the 4-stage experimental 2D differentiation protocol for the generation of SOX9^+^ progenitors. **C** GFP2 expression during differentiation of the SC30 and NSC20 cells. Data are means ± SEM, n = 4–8. Arrows mark developmental stages. **D** Flow cytometry analysis of GFP2 and H2-K^k^ expression in GFP2^±^ cells at d12. **E** RT-qPCR analysis of sorted SC30 derived GFP2^+^ and GFP2^−^ cells (d11-d12). Depicted is the relative gene expression of *HNF6*, *NKX6-1*, *PDX1*, *SOX9* and *NEUROG3*. Data are means ± SEM, n = 3–4. Two-tailed *Student*’*s* t-test. **F** IF staining of SOX9 (red) in SC30 derived GFP2^+^ and GFP2^−^ cells (d12). Nuclei were counterstained with DAPI. **G** IF staining of PDX1, HNF-6 and NKX6-1 (red) and FOXA2 (green) of sorted SC30 derived GFP2^+^ cells (d12). **F**/**G** Scale bar = 100 µm. **H** Flow cytometry dot plot and histogram of SC30 GFP2 and CD200, CD142 and GP2 expression at d12 and d15 (end of stage 4). Bifurcated gates were set according to unstained controls, values are means ± SEM, n = 3–4

**Fig. 2:**
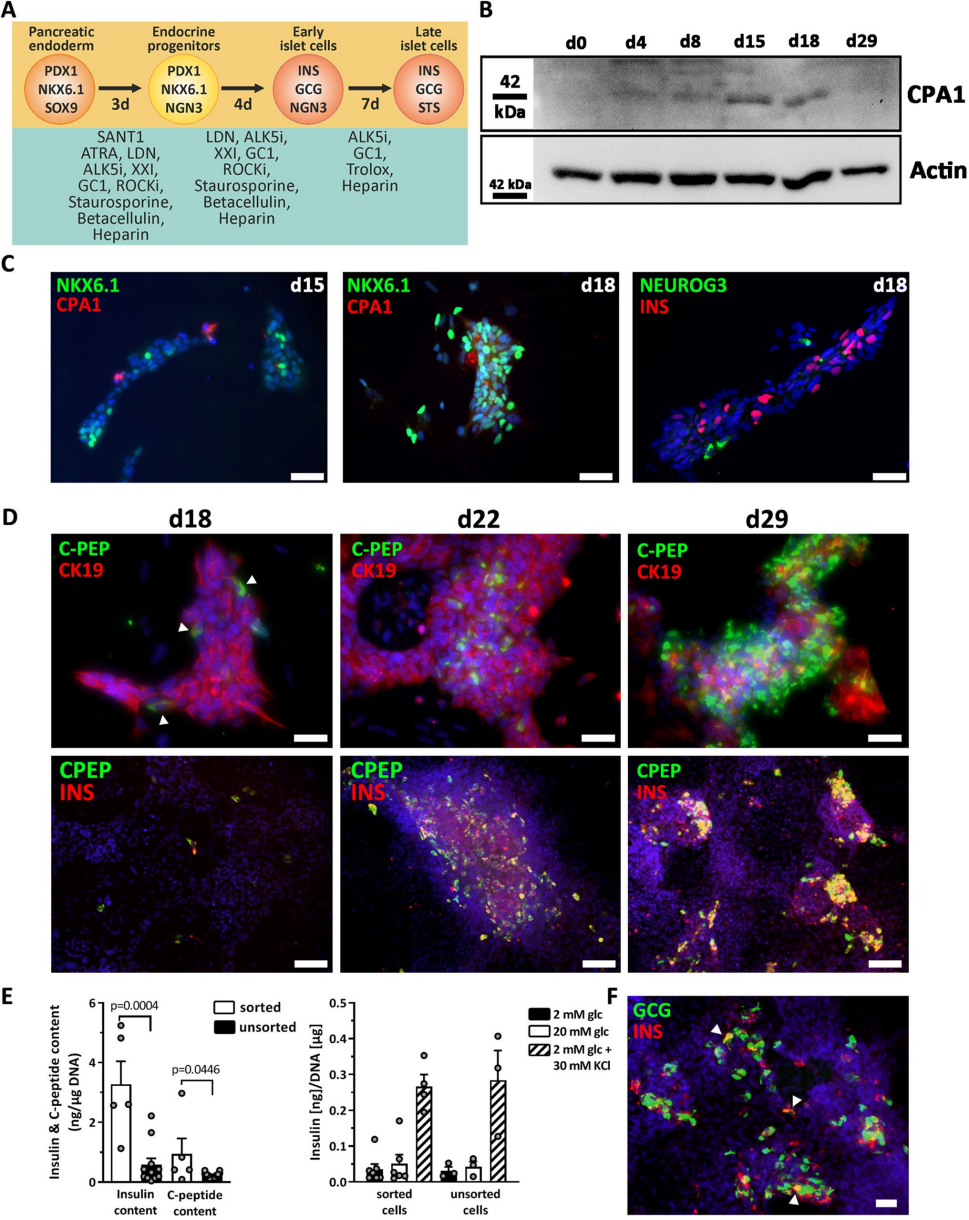
**A** Scheme of the 3-stage protocol for differentiation of SOX9^+^ MPCs into stem cell-derived beta cells. MACS was performed at d12 using the SC30 cell clone. **B** Analysis of CPA1 protein expression during differentiation (d0-d29) by Western Blot. **C** IF staining of NKX6.1/CPA1 and NEUROG3/insulin after stage 4 (d15) or 5 (d18), respectively. Scale bar = 50 µm. **D** IF staining of CK19/C-peptide and insulin/C-peptide at d18, d22 or d29 of differentiation. Scale bar = 50 µm (d18 CK19/C-peptide) or 100 µm. Arrowheads indicate early insulin-positive cells. **E** Measurement of insulin and C-peptide secretion and content in d29 sorted SC30 cells vs unsorted cells. Data are means ± SEM, n = 5–12 (content) and n = 3–7 (secretion). Two-tailed *Student*’*s* t-test. **F** IF staining of glucagon and insulin in d29 cells. Arrowheads mark polyhormonal cells. Scale bar = 50 µm

**Fig. 3:**
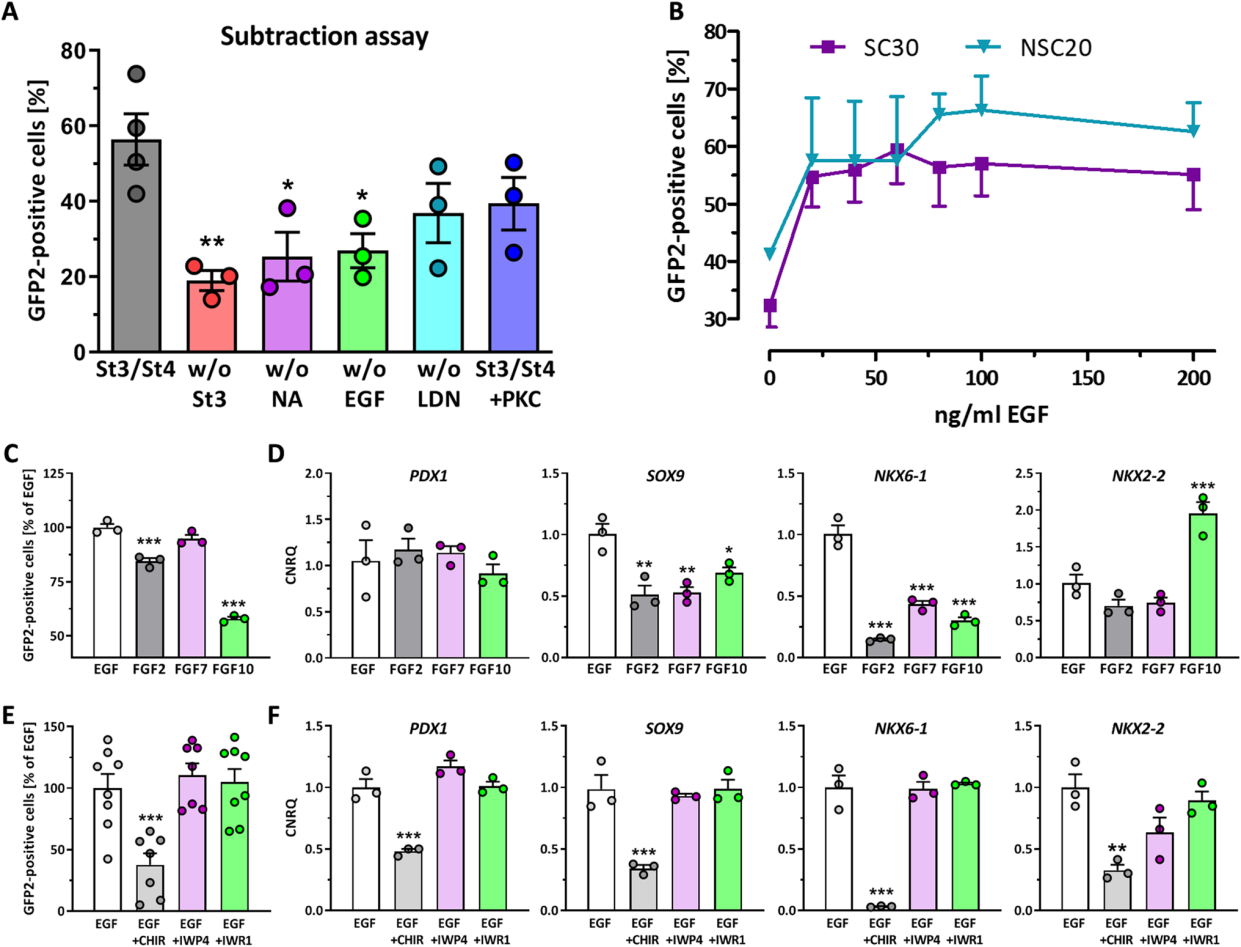
**A** Measurement of GFP2^+^ cells at d12 after subtraction of either stage 3, nicotinamide (NA), EGF, or LDN193189 (LDN). For comparison stage 4 plus protein kinase C activation by 100 nM PDBu. Data are means ± SEM, n = 3. **B** GFP2-expression in dependence of the EGF concentration at d12. Data are means ± SEM, n = 3. **C/D** Effect of different growth factors each used at 100 ng/ml on GFP2 expression (**C**) at d12 and pancreatic marker gene expression (**D**). Depicted is the relative gene expression of *PDX1*, *SOX9*, *NKX6-1*, and *NKX2-2*. Data are means ± SEM. n = 7–8 (GFP2 flow cytometry), n = 3 (RT-qPCR). (E/F) Effect of canonical Wnt-signaling on GFP2 expression (**E**) at d12 and pancreatic marker gene expression (**F**). The pathway was activated by CHIR (3 µM) or inhibited by IWP4 (1 µM) or IWR-1 (2 µM). Depicted is the relative gene expression of *PDX1*, *SOX9*, *NKX6-1*, and *NKX2-2*. Data are means ± SEM. n = 3 (GFP2, RT-qPCR). ANOVA plus *Dunnett*’*s* post-test, ***p < 0.001, **p < 0.01, *p < 0.05

**Fig. 4:**
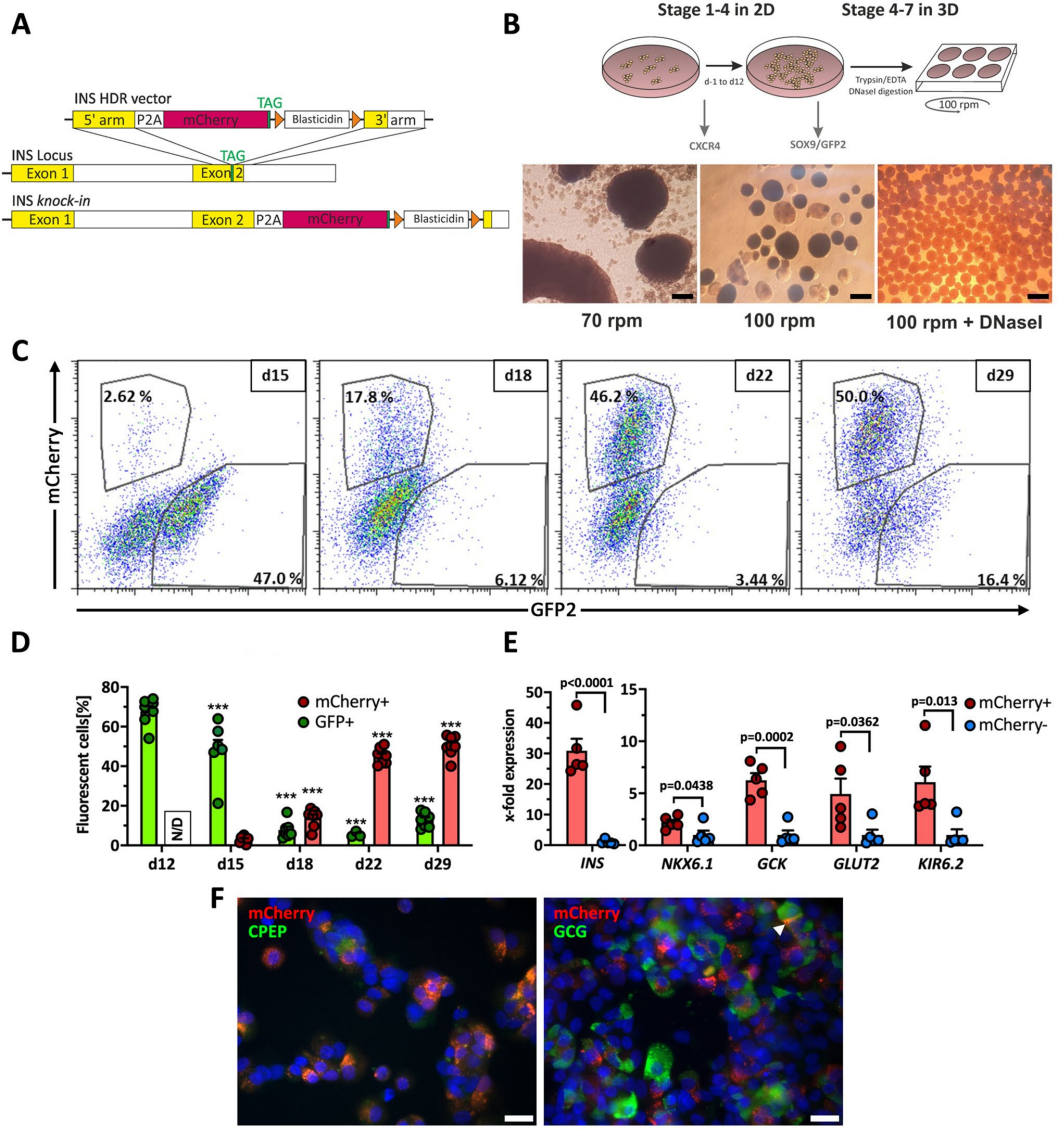
**A** Scheme of the INS HDR vector and the human *INS*-locus before and after HDR. **B** Scheme of the 3D production protocol for the differentiation of SC30/NSC20 and SC30 ICNC4 cell clones into stem cell-derived islets and phase contrast images of clusters generated by 3D orbital shaking culture. **C** Flow cytometry dot plots of mCherry vs GFP2 expression during 3D differentiation. Gate numbers indicate the [%] of SOX9- or INS-expressing cells. Scale bar = 500 µm. **D** Kinetics of GFP2 and mCherry protein expression in SC30 ICNC4 cells during 3D differentiation. Values are means ± SEM. n = 3–7. ANOVA plus *Tukey*’*s* post-test, ***p < 0.001, **p < 0.01, *p < 0.05, compared to d12/d15 of differentiation. **E** RT-qPCR analysis of the beta cell genes *INS*, *NKX6-1*, glucokinase (*GCK*), *GLUT2* (*SLC2A2*) and *KIR6.2* (*KCNJ11*) in sorted mCherry^+^ vs mCherry^−^ cells at d29 of differentiation generated with the SC30 ICNC4 cell clone. Values are means ± SEM. n = 5, two-tailed *Student*’*s* t-test. Expression values for mCherry^−^ cells were set to 1. **F** IF staining of C-peptide (left) and glucagon (right) overlayed with the native mCherry fluorescence in d29 unsorted SC30 ICNC4 cells on glass slides. Scale bar = 20 µm. The arrowhead marks a polyhormonal cells

**Fig. 5:**
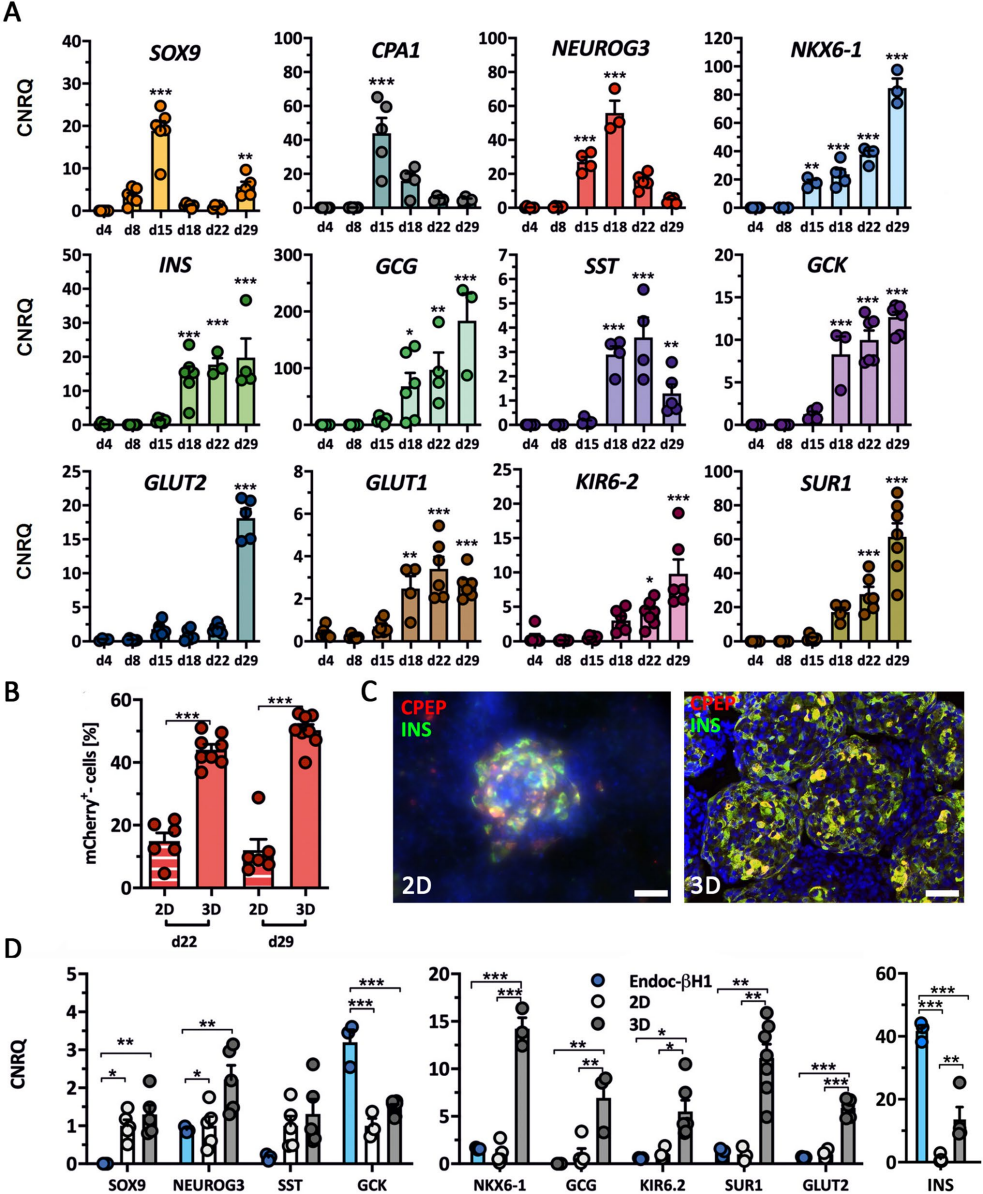
**A** Expression kinetics of pancreatic and endocrine genes during differentiation from d4 to d29 measured by RT-qPCR in SC30 ICNC4 cells. Values are means ± SEM. n = 3–6. ANOVA plus *Tukey*’*s* post-test, ***p < 0.001, **p < 0.01, *p < 0.05, compared to d4 of differentiation. **B** Effect of 2D vs 3D differentiation on mCherry expression at d22 and d29 in SC30 ICNC4 cells. Values are means ± SEM. n = 4–6. ANOVA plus *Tukey*’*s* post-test, ***p < 0.001, **p < 0.01, *p < 0.05. **C** Double-IF staining of insulin (green) and C-peptide (red) in SC30-derived cells at d29 in 2D- or 3D-derived cells. Scale bar = 50 µm. **D** Effect of 2D vs 3D differentiation on pancreatic and endocrine marker gene expression measured by RT-qPCR comparing SC30 ICNC4 to EndoC-βH1 cells. Values are means ± SEM. n = 3–6. ANOVA plus *Tukey*’*s* post-test, ***p < 0.001, **p < 0.01, *p < 0.05 compared to d4. Relative expression values for 2D differentiation were set to 1

**Fig. 6:**
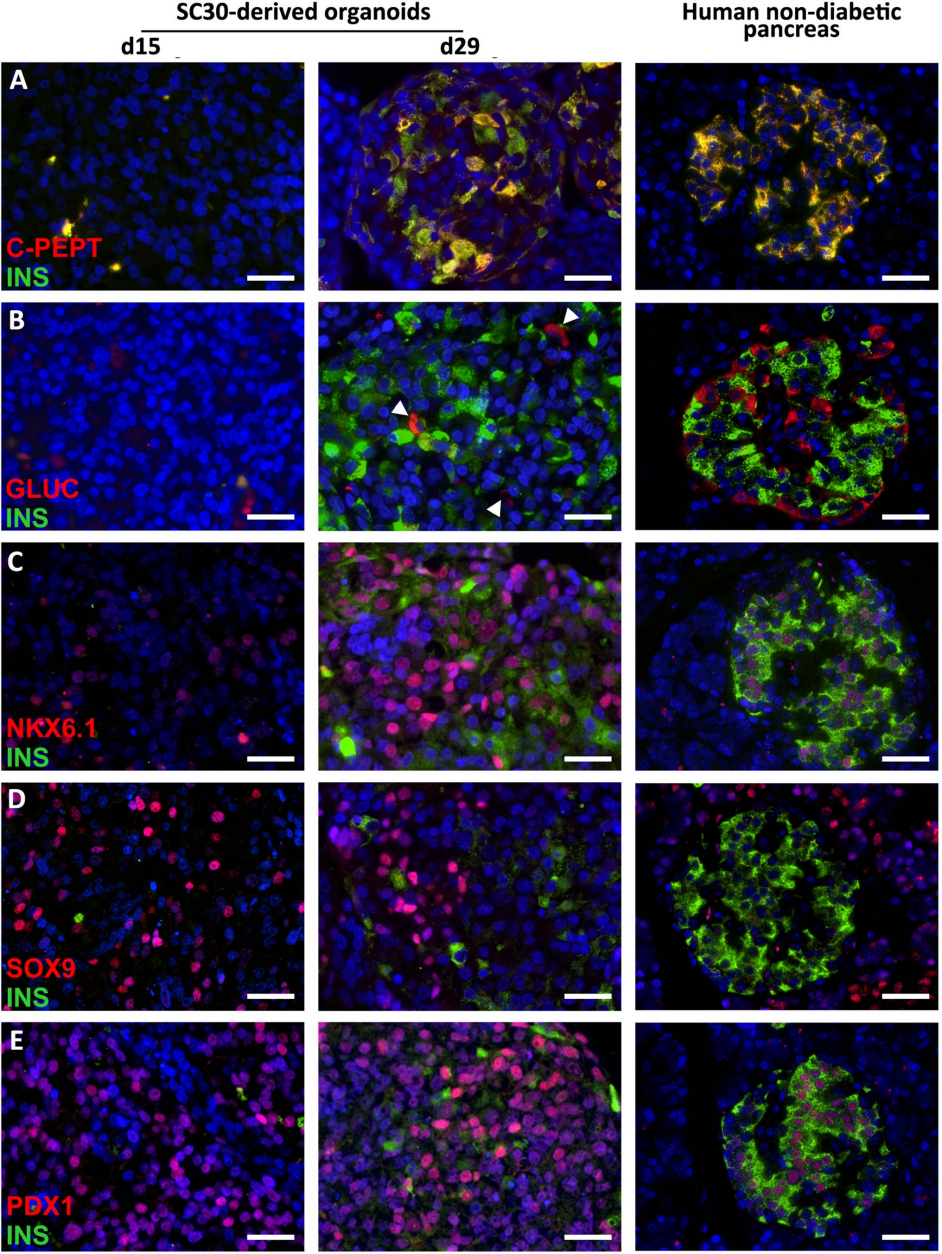
Immunhistochemical analysis of SC-derived pancreatic organoids. d15 spheroids and d29 stem cell-derived organoids derived in 3D from the SC30 clone were fixed, sectioned and double-stained for (**A**) C-peptide (red) and insulin (green) or insulin (green) and glucagon, NKX6.1, SOX9 or PDX1 (**B-E**, all in red). A human non-diabetic pancreas was taken as control. Scale bars = 20 µm

**Fig. 7:**
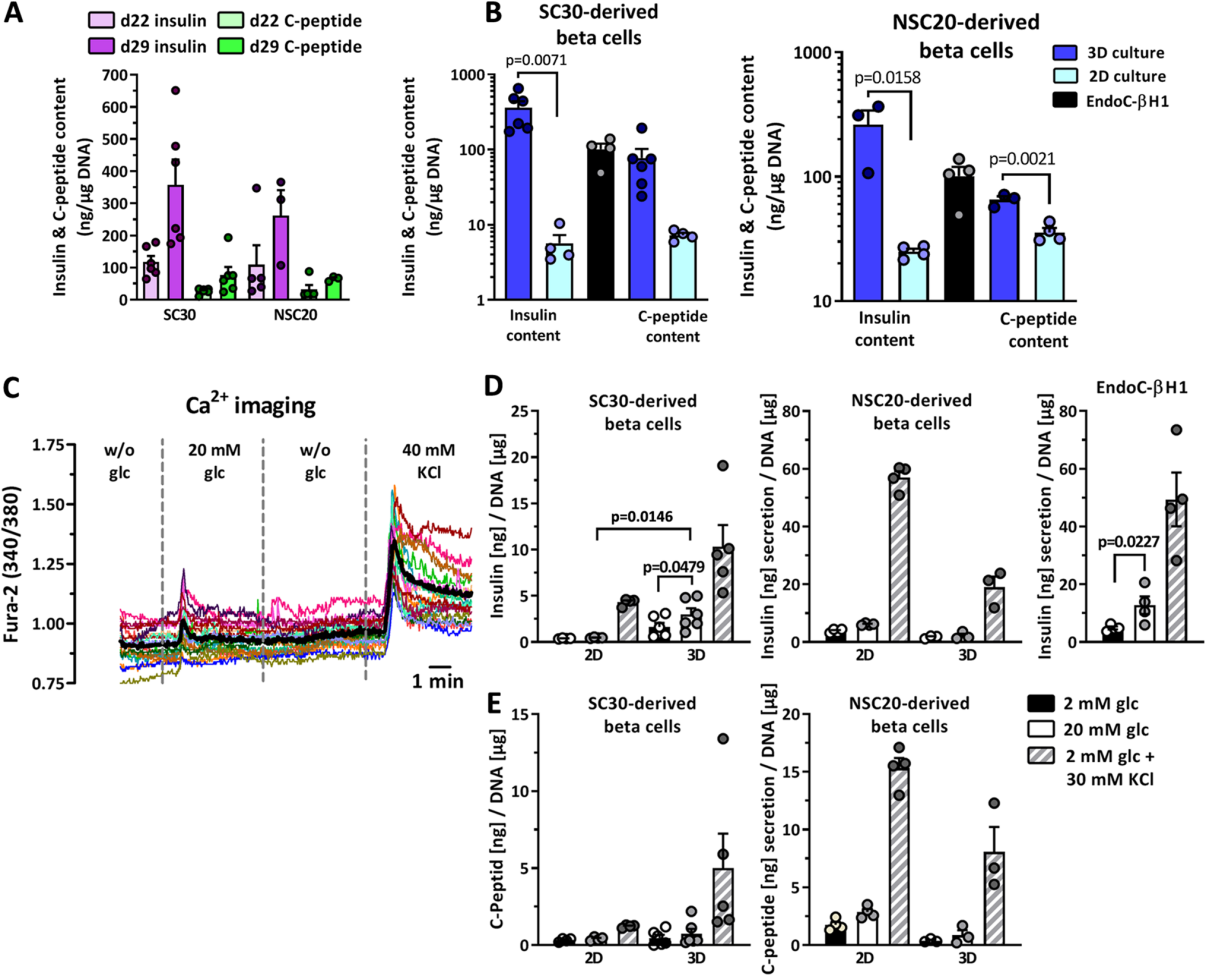
**A** Insulin and C-peptide content of NSC20- and SC30-derived organoids at d22 and d29 of differentiation in 3D. Values are means ± SEM, n = 3–6. **B** Insulin and C-peptide content of NSC20- and SC30-derived organoids at d29 differentiated in 2D or 3D in comparison to EndoC-βH1 cells. Two-tailed *Student*’*s* t-test. **C** Real time detection of cytosolic free-Ca^2+^ in SC30-derived organoids by recording of the Fura-2/AM emission ratio at 340 and 380 nm. The cells were perifused with basal KR w/o glucose, 20 mM glucose in KR, basal KR w/o glucose and finally KR plus 40 mM KCl. Mean value of 19 recorded cells are shown in bold black. **D/E** Measurement of insulin and C-peptide secretion in NSC20- and SC30-derived organoids at d29 after 2D and 3D differentiation in comparison to EndoC-βH1 cells. Data are means ± SEM, n = 3–6. Two-tailed *Student*’*s* t-test

To introduce DNA double-strand breaks (DSBs) into the loci of *SOX9* and *INS,* CRISPR/Cas9 were used. SgRNAs were calculated with CCTop [14]. The sgRNA for the *SOX9*-locus was cloned into the pLKO5.U6 vector [15] and sgRNAs, two nickase pairs, for the *INS*-locus were cloned into the pX335-U6-Chimeric_BB-CBh-hSpCas9n vector [16]. SgRNA sequence details are available in Supplementary table 1. Reporter genes were introduced by HDR [17]. A scheme of the repair vectors is depicted in Figs. 1A/4A. Briefly 2 × 10^6^ hPSCs were nucleofected with 2 µg repair vector and 2 µg Cas9/sgRNA vector (Neon Nucleofection System). Transfected hPSCs were seeded and selected in mTeSR1 medium after 24 h using hygromycin B or blasticidin. Cell clones were picked after 10–14 days, expanded in mTeSR1 medium, PCR-genotyped and sequenced upon correct insertion. To detect the majority of karyotypic abnormalities reported in hPSCs, a genetic analysis kit was used (Stem Cell Technologies, see Supplementary Karyotype Report). HES-3 SC30 ICNC4 cells were used to optimize the 3D differentiation protocol (data in Figs. 4, 5), whereas HES-3 SC30 cells (with an unchanged *INS*-locus) were used to characterize the beta-cell phenotype and optimize the differentiation towards SOX9 MPCs (data in Figs. 1, 2, 3 and 6, 7). 

### 2D Experimental Differentiation

Differentiation experiments were performed at 37 °C and 5% CO_2_. For differentiation in 2D, hPSC colonies were dissociated into single cells by 0.05% Trypsin/0.02% EDTA (T/E) (Biochrom, Berlin, Germany) and centrifuged for 3 min at 300×*g*. The pellet was re-suspended in mTeSR1/iPS-Brew containing 5 µM Y-27632 (Selleck Chemicals, Munich, Germany) and a defined number of cells (100,000 cells/12-well plate cavity, 250,000 cells/6-well plate cavity and 1.3–1.45 × 10^6^ cells per 10 cm cell culture dish) were seeded on Matrigel-coated cell culture plastics. Differentiation was initiated after 24 h and performed according to an adopted 7-stage protocol (Fig. 1B/2A) [10, 11, 18–20]. Compositions for stage 1- stage 7 media and suppliers are available online (Supplementary Methods). The required activin A (Stem Cell Technologies) concentration was titrated beforehand (Supplementary Fig. 2A). FGF2, FGF7 and FGF10 were used in the concentration of 100 ng/ml and IWP4 at 1 µM (Stem Cell Technologies). The concentration of the PKC activator PDBu was 100 nM (Bio-Techne, Minneapolis, MN, USA).

### 3D Production Protocol

The 3D differentiation protocol was initiated in 2D on Matrigel-coated cell culture plastic. Media compositions remained the same as described for 2D with slight differences. Stage 3 and stage 4 medium were supplemented with 2 µM IWR-1 (Selleck Chemicals) and 100 ng/ml EGF (Stem Cell Technologies) was supplemented to stage 4 medium. On day 12 of differentiation the cells were washed with PBS (Sigma-Aldrich, Munich, Germany), dissociated by T/E and centrifuged for 4 min at 300×*g*. The cell pellet was re-suspended in 0.1 mg/ml DNaseI (Sigma-Aldrich) in PBS + 10% FCS (PAA, Vienna, Austria) and incubated at room temperature for 15–20 min. Then the cells were centrifuged for 4 min at 300×*g* and re-suspended in stage 4 medium supplemented with 5 µM Y-27632. 2 × 10^6^ cells/ml were inoculated on a 6-well suspension culture plate (Greiner Bio-One, Kremsmünster, Austria) and cultivated at 100 rpm (Infors HT, Celltron, Bottmingen, Switzerland).

### Western Blot

Cells at d0, d4, d8, d15, d18 and d29 were harvested in PBS and sonified for 30 s/60 Amplitude % on a Braun-Sonic 125 homogenisator (Quigley-Rochester Inc., Rochester, NY, USA). A protease inhibitor mixture (Roche Diagnostic, Mannheim, Germany) was added. Protein content was determined via BCA (Thermo Fisher Scientific, Schwerte, Germany). 40 µg protein was loaded and separated by SDS-PAGE (10%) and transferred by electro-blotting to PVDF membranes. Blocking was performed with 5% nonfat dry milk in PBS plus 0.1% Tween 20. Membranes were incubated with anti-CPA1 (1:1000, OriGene Technologies, Herford, Germany, cat# TA500053, clone OTI2A3) overnight at 4 °C, washed, followed by incubation with the peroxidase-labeled secondary antibody for 1 h. As a loading control actin was used. Protein bands were visualized by an ECL detection kit (GE Healthcare Europe, Solingen, Germany) on a chemiluminescence imager (INTAS Science imaging, Göttingen, Germany).

### Gene Expression Analysis

Isolation of total RNA was done with the Machery&Nagel Nucleospin RNA plus Kit (Macherey&Nagel, Düren, Germany). cDNA was synthesized from 500 to 2000 ng total RNA using RevertAid™ H Minus M-MuLV RT (Thermo Fisher Scientific) and random hexamer primers (Thermo Fisher Scientific). cDNA samples were diluted to 2.5–5 ng/µl (the cDNA concentration was extrapolated from the measured RNA concentration) and measured in qPCR reactions with the GoTaq® qPCR Master Mix (Promega, Walldorf, Germany). A 2-step PCR in triplicates followed by melting curve analysis on a ViiA7 real-time PCR cycler was performed (Thermo Fisher Scientific). Primers are specified in supplementary table 2. Data normalization was performed with qBasePlus (Biogazelle, Zwijnaarde, Belgium) against the housekeeping genes *G6PD*, *TBP* and *TUBA1A*. RT-qPCR data are presented as calibrated normalized relative quantities (CNRQ).

### Flow Cytometry

Cells were washed with PBS and dissociated using T/E. Organoids were collected in a 15 ml conical tube, centrifuged at 50×*g* for 5 min and subsequently dissociated with gentle cell dissociation solution (Stem Cell Technologies) for 15 min and additional T/E for 10 min. Single cells were then centrifuged at 300×*g* for 3 min and re-suspended in PBS + 2% FCS before flow cytometric measurement. For flow cytometric staining 1 × 10^6^ cells were washed, incubated for 20 min at 4 °C with primary conjugated antibodies and washed twice prior to analysis. Flow cytometry was performed on a CyFlow ML flow cytometer (Partec, Münster, Germany). Data analysis was performed using the FlowJo software (Ashland, OR, USA).

### Cell Sorting

Fluorescence activated cell sorting (FACS) was performed at the central facility of the Hannover Medical School. For MACS 1 × 10^7^ dissociated cells were harvested in PBE buffer (PBS, pH 7.2, 0.5% BSA, 2 mM EDTA) and were then conjugated with anti H2-K^k^ magnetic microbeads (Miltenyi-Biotec) for 15 min on ice and sorted with an autoMACS Pro (Miltenyi-Biotec).

### Immunofluorescence

For immunofluorescence (IF) staining, hPSCs were seeded onto slides (SPL Life Sciences, Pocheon, South Korea) with 5 µM Y-27632. After 24 h the cells were fixated with 4% (w/v) paraformaldehyde (PFA, Sigma-Aldrich), buffered in PBS, pH 7.4. The same fixation was used for organoids at day 15 and day 29 which were embedded in paraffin and sectioned. After pretreatment and blocking steps the same primary antibodies were used for cells and organoids and incubated for 1–3 h at room temperature or overnight at 4 °C (Supplementary Table 3). Cells and organoids were stained with AlexaFluor/Cy-conjugated secondary antibodies (Dianova, Hamburg, Germany) and embedded in mounting medium containing DAPI (Dianova). For comparison, immunostaining of human islets from four non-diabetic donors was performed (Supplementary Table 4). Stained cells/organoids were examined using an Olympus IX81 or Olympus BX61 microscope (Olympus, Hamburg, Germany). Pictures were analyzed as previously described [21].

### Insulin and C-Peptide Content and Secretion

Cells grown in 2D/3D culture were washed with Krebs–Ringer (KR) solution and starved for 2 h in KR without glucose plus 0.1% albumin. Thereafter the cells were stimulated with 2 or 20 mM glucose or 2 mM glucose and 30 mM KCl for 1 h. To measure insulin and C-peptide secretion, the medium supernatant was removed and centrifuged for 5 min at 700×*g*. The cells were reconstituted in PBS, sonicated and centrifuged for 5 min at 700×*g*. Secreted insulin in the supernatant and insulin content of the incubated cells were determined by radioimmunoassay and the resulting values were normalized to DNA content [13]. C-peptide content and C-peptide secretion was measured by an ELISA assay (DRG Diagnostics, Marburg, Germany).

### Calcium Imaging

Cytosolic free-Ca^2+^ was determined with Fura-2/AM. D28 clusters were dissociated, grown overnight on glass slides and loaded with 3 μM Fura-2/AM by incubation in modified Krebs–Ringer (KR) solution (25 mM HEPES, 3 mM glucose, and 1.5% BSA) for 30 min at 37 °C. Perifusion was performed with 0 mM or 20 mM glucose and 0 mM glucose plus 40 mM KCl at a flow rate of 1 ml/min using a peristaltic pump (Ismatec, Zürich, Switzerland). Images were taken every 2 s using the inverted IX81 microscope equipped with an incubation chamber to maintain 60% humidity, 37 °C, and 5% CO_2_.

### Statistics

Unless stated otherwise, values represent mean ± SEM and the number of independent experiments (n) is stated in each figure legend along with the statistical test performed. Statistical analyses were done with GraphPad Prism analysis software (Graphpad, San Diego, CA, USA) using unpaired, two-tailed *Student*’*s *t-test or ANOVA plus *Dunnett*’*s* or *Tukey*’*s* post-hoc tests for multiple comparisons. P-values for *Student*’*s* t-test are depicted in each figure.

## Results

To generate reporter PSC lines, a knock-in strategy based on CRISPR/Cas9-induced DSBs was developed (Supplementary Table 1). The repair vector comprised 500 bp 5′ and 3′ homology arms, the genes H-2K^k^ and GFP2, separated by 2A cleavage sites, and a floxed selection gene (Fig. 1A). After nucleofection and selection, pluripotent stem cell colonies with typical morphology were expanded and genotyped by PCR. Correct insertion was verified by sequencing. The HES-3 clone SC30 and the Phoenix clones NSC19/NSC20, all with homozygous integration, were selected for further work. Cell clones expressed typical levels of pluripotency transcription factors and surface antigens (Supplementary Fig. 1). An analysis of the most common karyotype changes in hPSC showed that the parental lines and the cell clones had amplifications on chromosome 20, which is typically found in more than 20% of hPSC lines worldwide [22, 23] (Supplementary karyotype report).

Our endoderm differentiation protocol [18] robustly produced > 90% CXCR4^+^ cells. CXCR4^+^ cells were also predominantly positive for the anterior foregut endoderm markers CD177 and CD275 as recently described [24] (Supplementary Fig. 2A–D). Then six different protocols for differentiation were tested upon their ability to induce expression of typical MPC marker genes (Supplementary Fig. 3). Protocol # 6, which is based on our protocol for the differentiation of hPSCs into PDX1^+^ pancreatic-duodenal cells (Supplementary Fig. 2E) [10, 11] expanded by adopted Stage 3 and Stage 4 media from the adapted protocol [19] was then selected and termed 2D experimental differentiation protocol (Fig. 1B). This 4-stage protocol yielded in an expression peak of ~ 40–50% GFP2^+^ cells between day 10 and 13 of differentiation (Fig. 1C). GFP2^+^ cells additionally expressed H-2K^k^, thereby allowing cell purification by either FACS or MACS (Fig. 1D/Supplementary Fig. 4).

### Knock-In of H-2K^k^ and GFP2 into the SOX9-Locus Enables Cell Sorting of MPC

Next, GFP2^+^/GFP2^−^ cells were sorted by FACS and analyzed upon expression of MPC genes. GFP2^+^ cells expressed significantly more *HNF6*, *NKX6-1*, *PDX1* and *SOX9* compared to GFP2^−^ cells. Only *NEUROG3* was stronger expressed in GFP^−^ cells (Fig. 1E/Supplementary Fig. 5).

SOX9 predominately occurred in the nucleus of GFP2^+^ cells and was low or absent in GFP2^−^ cells (Fig. 1F). Consistently, PDX1, NKX6-1 and HNF6 were expressed in GFP2^+^ cells (Fig. 1G). Next, MPC surface markers, namely CD200 and CD142 [25] and GP2 [26], were measured by flow cytometry. D12 and d15 GFP2^+^ cells expressed CD200 and GP2 and faintly CD142, but only GP2 was differently expressed compared to GFP^−^ cells (Fig. 1H).

According to the expression pattern of Sox9 during pancreas organogenesis in mice, we expected to detect CPA1^+^/SOX9^+^ and NKX6.1^+^/SOX9^+^ cells as representatives of distal and proximal tip/trunk cells in this differentiation model. Western blot analysis showed a peak CPA1 expression in d15/d18 cells. To confirm this, we used MACS to purify SOX9 MPC and further differentiated them in 2D using the stage 5–7 media previously described [20]. Purified SOX9 MPC formed tight colonies after re-seeding. At d15 NKX6.1 and CPA1 expressing cells in a mosaic-like pattern were detected and by d18 NKX6.1 was almost homogenously expressed in these colonies. D18 marked also the expression peak of scattered NEUROG3^+^ and very few CPA1^+^ cells (Fig. 2C). The detection of the first NEUROG3^+^ cells coincided with insulin^+^ and C-peptide^+^ cells, which from d18 grew out in clusters. These structures were embedded in epithelial-like CK19^+^ colonies (Fig. 2D). Gene expression changes of sorted and further differentiated cells are presented in supplementary Fig. 6. We next analyzed insulin and C-peptide content, to address if these SC-derived beta cells release insulin in response to glucose and compared sorted vs non-sorted cultures. Cell sorting yielded in a ~ 4-fold increased insulin and C-peptide content compared to non-sorted cells (Fig. 2E). However, insulin release was only triggered by KCl and not by elevated glucose concentrations (Fig. 2E). Differentiation of hPSCs into polyhormonal SC-beta cells is a common phenomenon. Thus, we stained for insulin and glucagon and found low numbers of polyhormonal cells (Fig. 2F).

### EGF-Signaling is Effective in Generating MPC

Next, to take advantage of the SOX9 reporter cells, we tested the impact of stage 3 and stage 4 media components. In a subtraction assay we compared GFP2 expression in controls cultured for 24 h in stage 3 and 72 h in stage 4 against treatments, where stage 3 was omitted or individual components of stage 4 were left out (Fig. 3A). The omission of stage 3 showed the greatest and significant reduction of GFP2^+^ cells. In descending order, the number of GFP2^+^ cells was reduced by omitting LDN, EGF and NA from stage 4 medium. Addition of a PKC activator as used in some differentiation protocols, however, did not increase the number of GFP2^+^ cells in our protocol. Then we titrated EGF in stage 4 medium and were able to determine that the maximum of GFP2 expression was reached for both cell lines at 100 ng/ml EGF (Fig. 3B). The SOX9 gene is controlled, amongst other mechanisms, by the FGFR2b signaling and by Wnt/beta-catenin signaling [27]. Therefore we compared the effects of the growth factors FGF7 and FGF10, which signals through FGFR2b, and of EGF and FGF2 used in other beta cell differentiation protocols at this differentiation stage [19, 20, 26, 28].

### Wnt/Beta-Catenin Signaling is a Strong Inhibitor of MPC Development

The effect of Wnt/beta-catenin inhibition and activation was also analyzed (Fig. 3C–F). The incubation with 100 ng/ml EGF produced the highest number of GFP2^+^ cells and showed the strongest expression of *PDX1*, *SOX9* and *NKX6-1*. FGF10 and FGF2 at the same concentration caused a decrease of GFP2^+^ cells compared to EGF (Fig. 3C). Wnt/beta-catenin activation of this pathway by CHIR99021 showed a significant inhibitory effect on the number of GFP2^+^ cells and on the expression of *PDX1*, *SOX9*, *NKX6-1* and *NKX2-2*. Addition of the Wnt/beta-catenin signaling inhibitors IWP4 and IWR-1, however, did not yield in an increase of GFP2^+^ cells or enhanced expression (Fig. 3E/F). The same pattern was found for NSC20 cells (Supplementary Fig. 7A/B). Furthermore, we were able to determine a significant increase in GFP2 expression from 42 to 74% for the SC30 cell clone and from 46 to 77% for the NSC20 cell clone after upscaling from 2D differentiation in 12-well plates to 10 cm culture dishes (Supplementary Fig. 7C).

### Knock-In of mCherry into the INS-Locus Enables Observation and Cell Sorting of SC-Beta Cells

For simplification of the monitoring process, we knocked-in mCherry into the human *INS*-locus of the SC30 cell clone. This allowed us to measure SOX9 MPC generation and their conversion into SC-derived beta cells. For this, a repair vector was cloned that comprised the reporter gene mCherry and a floxed selection gene (Fig. 4A). For the knock-in into the *INS*-locus, CRISPR/Cas9 nickases were used (Supplementary Table 1). The HES-3 SC30 ICNC4 cell clone with homozygous integration was selected for further work.

With reports on the improvement of differentiation through 3D culture we adapted our 2D experimental protocol and introduced 3D orbital shaking from d12 on until the end of differentiation (Stage 4–7). Stage 3 and 4 media were adapted according to our findings. For 3D differentiation, d12 cells were gently dissociated and transferred to 6-well suspension culture plates on an orbital shaker. Different rotating speeds from 70 to 100 rpm were tested. Finally, we inoculated 2 × 10^6^ cells per ml in 5 ml total with 100 rpm orbital shaking. Prior to seeding, the cells were treated with Dnase I, yielding in equally formed, round and small spheroids 24 h after transition from 2 to 3D culture. The spheroids were differentiated for an additional 16–17 days without resizing. (Fig. 4B, Supplementary Fig. 8).

During this time the kinetics of GFP2 and mCherry expression were monitored by flow cytometry. The increase in mCherry^+^ cells was accompanied by a parallel decrease in GFP2^+^/SOX9^+^ cells starting from d15 (Fig. 4C/D). GFP2^+^/mCherry^+^ cells were rarely seen. Next, we verified the *INS* knock-in by comparing expression of beta cell genes in mCherry^+^ vs mCherry^−^ cells. *INS* expression was 31-fold higher in mCherry^+^ cells and other beta cell markers were between 2.2- and 6.1-fold higher expressed compared to mCherry^−^ cells (Fig. 4E). IF staining of C-peptide showed a clear co-localization with mCherry verifying functionality of the knock-in (Fig. 4F). Polyhormonal cells were again rarely detected (Fig. 4F).

### Differentiation in 3D Generates Pancreatic Organoids with a Beta Cell Content of > 40%

The expression kinetics of pancreatic/endocrine genes was monitored during the 2D/3D differentiation process (Fig. 5A). Of note here was again peak expression of *SOX9* and *CPA1* at d15 followed by peak *NEUROG3* expression. In parallel with *NEUROG3* expression, the increase in islet cell hormones and *NKX6-1* could be recorded. Typical beta cell genes showed the same pattern in gene expression as insulin (Fig. 5A).

Next, the number of mCherry^+^/*INS*^+^ cells obtained in 2D culture was compared to 3D culture. 3D yielded in significantly more mCherry^+^/*INS*^+^ cells compared to 2D differentiation (mean 44% for d22 in 3D, 15% for d22 in 2D; mean 50% for d29 in 3D, 12% for d29 in 2D) (Fig. 5B). The different efficiencies of 2D and 3D differentiation could also be observed after immunofluorescence (IF) staining of insulin and C-peptide (Fig. 5C). The expression of glucose recognition marker genes *GCK*, *KIR6.2*, *SUR1* and *GLUT2* and the transcription factor *NKX6-1* were significantly higher in 3D compared to 2D conditions and, except for *GCK* expression, comparable to EndoC-βH1 cells (Fig. 5D). Islet hormone expression was also higher in 3D. Since EndoC-βH1 cells are a beta cell model line, *INS* expression was higher compared to the heterogeneous composition of SC-derived organoids. The expression of the transcription factors *NEUROG3* and *SOX9* were also highest for 3D culture. This together with the gene expression analysis in 2D and 3D compared to EndoC-βH1 cells revealed the improvement of differentiation towards SC-derived organoids in 3D culture (Fig. 5D/Supplementary Fig. 9). SC30-derived organoids from d15 and d29 were then compared to human pancreatic tissue from non-diabetic pancreases by IF (Fig. 6). D29 SC-derived organoids generated from the SC30 cell clone were typically 200–300 µm in diameter and displayed a cytoplasmic co-localization of insulin and C-peptide in the majority of cells resembling beta cells in human islets. A polyhormonal staining of insulin or C-peptide with other islet peptides was rarely detected (Fig. 6/Supplementary Fig. 10). Also the number of glucagon-positive cells was lower compared to 2D culture. NKX6.1 and PDX1 were in parallel localized in the nucleus of insulin-positive cells in d29 SC-derived organoids. In comparison with human tissue, SC-derived organoids contained more SOX9^+^ cells, whereas in human pancreas sections a clearer distinction between the SOX9^−^ endocrine islet and SOX9^+^ exocrine parenchyma was observed (Fig. 6). For comparison, IF staining of insulin, C-peptide and glucagon of SC30 and NSC20 cells in 2D culture is depicted in Supplementary Fig. 11. Next the SC-derived organoids were further characterized. First, we were able to measure a distinct increase in insulin and C-peptide content from d22 to d29 organoids (Fig. 7A). This content was comparable to EndoC-βH1 cells and significantly higher compared to 2D (5.7 vs 302.27 ng insulin/µg DNA for SC30, respectively) (Fig. 7B).

### 3D Differentiation Results in Glucose-Responsive SC-Beta Cells

Then the changes in free cytosolic Ca^2+^ were measured. SC-derived organoids derived from the SC30 cell clone showed a detectable increase in free cytosolic calcium after exposure to glucose and KCl (Fig. 7C), while the NSC20 cell clone only responded to KCl (Supplementary Fig. 12).

Finally, the question was addressed whether 3D culture and differentiation could also improve glucose-induced insulin secretion (GSIS) in both cell lines (Fig. 7D/E). SC30-derived organoids showed a significant increase in insulin release when subjected to 20 mM glucose in a static assay. The insulin-releasing properties were also significantly improved compared to 2D culture. NSC20-derived organoids showed a more robust insulin release in 2D culture though but neither in 2D or 3D the cells responded appropriately in response to glucose. EndoC-βH1 cells were measured as controls (Fig. 7E).

## Discussion

Here we report the generation and characterization of hPSC reporter lines with insertion of GFP2 and H-2K^k^ following the *SOX9* open reading frame and a double knock-in cell line with an additional mCherry knock-in into the *INS*-locus. The selected genes enable cell sorting of specific pancreatic populations using FACS and MACS. It is, however, important to note that small peptides from the 2A sites remain at the C-terminus of the altered proteins and could potentially affect their function.

Aided by these cell lines we established a new differentiation protocol geared towards SOX9 MPCs to drive hPSCs by 3D orbital shaking culture into SC-derived organoids with a beta cell content of ~ 50%. SOX9 reporter cells showed peak SOX9/GFP2 expression after 10–13 days of differentiation using an experimental 2D differentiation protocol. This protocol is based on previous publications by our group in which we established a robust method for generating NKX6.1^−^/ PDX1^+^ pancreatic-duodenal cells [10, 11, 29] as well as adopted stage 3/stage 4 media previously described [19].

After purifying the GFP2^+^/GFP2^−^ fractions, we could show that GFP2^+^ cells expressed high levels of *HNF6*, *NKX6-1*, *PDX*1 and *SOX9*, and represent MPCs. *NEUROG3* expression was rather low in GFP2^+^ cells likely attributed to the repression of Sox9 by Ngn3 as reported [30]. Analysis of MACS-purified SOX9 MPCs at d15 revealed mosaic-like expression of NKX6.1 and a few CPA1^+^ cells. Possibly these cells are representatives of the trunk region, which represents the niche structure for further endocrine development. Analysis of the MPC surface markers CD142, CD200 and GP2 revealed partial identity with MPCs generated with different protocols [25, 26].

By further differentiation following the protocol published by Pagliuca and colleagues [20], the cells showed not only a more homogeneous NKX6.1 expression on d18, but also scattered NEUROG3^+^ cells could be observed. Then SOX9 MPCs readily developed into insulin/C-peptide-positive and glucagon-positive cells embedded in CK19^+^ epithelial cells. This fits in with findings from earlier studies that CK19^+^ fetal epithelium marks a source for endocrine islets [31]. In line with other studies polyhormonal cells were readily detected in 2D [32]. The SC-derived beta cell fraction generated in 2D showed no increased insulin secretion after glucose stimulation and comparably low insulin content. Purification of SOX9 MPCs could not compensate for this deficit, although insulin and C-peptide content were increased, which confirms the effectiveness of an enrichment strategy [26].

Before the transition to 3D differentiation, we systematically tested various compounds and conditions in stage 3 and 4 to increase the number of SOX9 MPCs. We can confirm that a 24 h pulse with stage 3 medium, which contains high FGF10 and a SHH inhibitor, is decisive for the differentiation into SOX9 MPCs. Withdrawal of nicotinamide and EGF in stage 4 also greatly reduced the number of SOX9 MPCs [19]. Interestingly, addition of the PKC activator PDBu was of no help at this stage of differentiation [33].

SOX9 is maintained by Wnt/beta-catenin signaling [34], FGF-signaling via FGFR2b [35], Notch-signaling [30] and positive autoregulation [36]. Moreover, SOX9 and Wnt/beta-catenin form a regulatory loop and inhibit each other’s transcriptional activity. In chondrocytes SOX9 inhibits canonical Wnt-signaling by direct protein interaction with beta-catenin, yielding in inhibition and degradation of the protein [37, 38]. Vice versa Wnt/beta-catenin represses SOX9 gene expression in osteoblasts [39]. In our in vitro differentiation approach, we can show for both hPSC lines that active canonical Wnt-signaling is an inhibitor of SOX9 MPC generation. This underlines the importance of this signaling pathway for differentiation of hPSC into SC-derived beta cells. Wnt/beta-catenin signaling not only prevents development of endocrine progenitors [40], but also posteriorizes the foregut towards hindgut identity [10] and, as shown here, prevents the generation of SOX9 MPCs.

Surprisingly our data indicate that differentiation into SOX9 MPCs is most effective in presence of EGF and not the commonly used growth factors FGF2/7 or FGF10 [26, 28, 41, 42]. FGF7 and FGF10 were less effective in terms of absolute numbers of SOX9 MPCs or showed lower expression of MPC marker genes. Previously we had identified FGF2 as a repressor of development into PDX1 pancreatic-duodenal cells [11]. In this study SOX9 MPC generation was also slightly less effective when compared to EGF. This was additionally evident from the reduced expression of *SOX9* and *NKX6-1* in FGF2-treated MPCs. In contrast to mouse studies for which an FGF10/FGFR2B/SOX9 feed-forward loop was described [35], the EGF-signaling pathway seems to play a greater role during differentiation into SOX9 MPCs in the human system. EGF was already used in a previous study although with the goal to maximize NKX6.1^+^/PDX1^+^ double-positive cells and not SOX9^+^ representatives of the trunk region [43].

3D differentiation in shaking orbital cultures or small bioreactors has become the standard for many somatic cell types [44–46]. Transition from 2D to 3D, without changes in extrinsic or other factors, can lead to a considerable phenotypic improvement [47]. In contrast to a 2D culture system, a 3D cell culture system can more precisely represent the actual microenvironment in which cells are located in tissues [48]. Therefore, differentiation of hPSC in 3D culture is thought to allow closer mimicry of in vivo development compared to 2D culture and thus differentiation protocols have improved using 3D culture [28, 47, 49]. With this in mind, a transition from a pure 2D protocol to a 2D/3D protocol was conducted for this study. At the same time, monitoring the differentiation with molecular typical biological methods such is time-consuming, cumbersome and uneconomical. In parallel, another knock-in was carried out into the *INS*-locus to be able to monitor differentiation efficiency changes by flow cytometry and to follow the conversion of SOX9 MPCs into SC-derived beta cells. The 3D islet-like organoids comprised SC-derived beta cells with important beta cell features such as expression of typical genes, insulin/C-peptide positive cells, very few polyhormonal cells, calcium influx after glucose exposure, and glucose-stimulated insulin secretion. Our results also show that transition from 2D to 3D culture not only results in a quantitative advantage, but also in a qualitatively improvement. It is also important to note that we observed line- and clone-specific effects. While the hESC-based clone differentiated into glucose-responsive cells, this was not achieved for the iPSC-based clone. This result is probably attributed to cell line-specific barriers, which have already been described and represent a major obstacle in the establishment of patient-specific cell replacement therapies [50, 51]. The clone ICNC4 also showed a higher tendency towards alpha cell differentiation than the parental SC30 line.

The currently prevailing differentiation protocols were optimized towards the generation of PDX1^+^/NKX6.1^+^ double-positive cells as the seed for SC-derived beta cells [20, 28]. Our protocol is based on an efficient generation of definitive endoderm using low activin A concentrations, differentiation into PDX1^+^ pancreatic-duodenal cells by BMP/Wnt-inhibition and all-trans retinoic acid signaling [10], and optimized conditions to generate around 70% SOX9 MPCs. SOX9 MPCs then effectively differentiate into SC-beta cells using previously reported conditions [20]. Thus, the protocol that we present in this report may offer an alternative route to generate SC-derived beta cells.

Reporter cell lines are excellent tools to advance research into efficient differentiation methods [52–55]. The SOX9/INS reporter cell lines reported here are excellent models for studying the in vitro differentiation of human bipotent ductal/endocrine precursors into insulin-producing cells and permit cell sorting. Single-pass cell sorting by FACS or MACS did, however, not yield in 100% pure populations and may need further optimization.

In view of the pleiotropic functions of SOX9 during development and tissue maintenance in diverse organs such as chondrocytes, testes, heart, lung, bile duct, retina and the central nervous system [37, 56, 57], these reporter cell lines are also suited for research on other matters. The reporter gene knock-in into the *INS*-locus enables an exclusive look at insulin-producing cells and can therefore bypass the problem of the heterogenous composition of in vitro differentiated cells caused by not yet fully effective differentiation protocols.

## Supplementary Information

Below is the link to the electronic supplementary material.

Supplementary file1 (DOCX 25 kb)

Supplementary file2 (DOCX 3.56 mb)

Supplementary file3 (DOCX 105 kb)

## Data Availability

The authors confirm that the data supporting the findings of this study are available within the article [and/or] its supplementary materials.

## Code Availability

Nothing to declare.
