# Supplementary material for: New hPSC SOX9 and INS Reporter Cell Lines Facilitate the Observation and Optimization of Differentiation into Insulin-Producing Cells
Source: Stem Cell Rev Rep. 2021 Aug 19;17(6):2193–209. doi: 10.1007/s12015-021-10232-9 (PMC8599335; doi:10.1007/s12015-021-10232-9)
Supplement: Supplementary file 1 — Supplementary file1 (DOCX 25 kb) [file 12015_2021_10232_MOESM1_ESM.docx]

**Supplementary methods**

**2D experimental protocol**

| **Stage 1A medium (24 h, day 0-1)** | **Final concentration** | **Supplier** | **Country of origin** |
| --- | --- | --- | --- |
| RPMI 1640 medium | NA | Biochrom | Berlin, Germany |
| 50x B27 supplement | 0.5x | Thermo Fisher Scientific | Schwerte, Germany |
| Penicillin/streptomycin | 1x | Santa Cruz Biotechnology/ Sigma-Aldrich | Dallas, Texas, USA/Munich, Germany |
| 200 mM glutamine | 2 mM | Sigma-Aldrich | Munich, Germany |
| 100x non-essential amino acids | 1x | Thermo Fisher Scientific | Schwerte, Germany |
| 100 mM So-pyruvate | 1 mM | Capricorn Scientific | Ebsdorfergrund, Germany |
| 100x ITS-X | 0.5x | Thermo Fisher Scientific | Schwerte, Germany |
| Vitamin C | 0.25 mM | Sigma-Aldrich | Munich, Germany |
| Activin A | 30 ng/ml | Stem Cell Technologies | Cologne, Germany |
| CHIR99021 | 3 µM | Cayman Chemical | Ann Arbor, Michigan, USA |

| **Stage 1B medium (72 h, day 1-4)** | **Final concentration** | **Supplier** | **Country of origin** |
| --- | --- | --- | --- |
| RPMI 1640 medium | NA | Biochrom | Berlin, Germany |
| 50x B27 supplement | 0.5x | Thermo Fisher Scientific | Schwerte, Germany |
| Penicillin/streptomycin | 1x | Santa Cruz Biotechnology/ Sigma-Aldrich | Dallas, Texas, USA/Munich, Germany |
| 200 mM glutamine | 2 mM | Sigma-Aldrich | Munich, Germany |
| 100x non-essential amino acids | 1x | Thermo Fisher Scientific | Schwerte, Germany |
| 100 mM So-pyruvate | 1 mM | Capricorn Scientific | Ebsdorfergrund, Germany |
| 100x ITS-X | 0.5x | Thermo Fisher Scientific | Schwerte, Germany |
| Vitamin C | 0.25 mM | Sigma-Aldrich | Munich, Germany |
| Activin A | 30 ng/ml | Stem Cell Technologies | Cologne, Germany |

| **Stage 2 medium (96 h, day 4-8)** | **Final concentration** | **Supplier** | **Country of origin** |
| --- | --- | --- | --- |
| Advanced RPMI 1640 medium | NA | Thermo Fisher Scientific | Schwerte, Germany |
| 50x B27 supplement | 0.5x | Thermo Fisher Scientific | Schwerte, Germany |
| Penicillin/streptomycin | 1x | Santa Cruz Biotechnology/ Sigma-Aldrich | Dallas, Texas, USA/Munich, Germany |
| 200 mM glutamine | 2 mM | Sigma-Aldrich | Munich, Germany |
| Vitamin C | 0.25 mM | Sigma-Aldrich | Munich, Germany |
| FGF7 | 5 ng/ml | Stem Cell Technologies | Cologne, Germany |
| IWR-1 | 2 µM | Selleck Chemicals | Munich, Germany |
| LDN193189 | 0.5 µM | Sigma-Aldrich | Munich, Germany |
| All-trans retinoic acid (ATRA) | 1 µM | Sigma-Aldrich | Munich, Germany |

| **Stage 3 medium (24 h, day 8-9)** | **Final concentration** | **Supplier** | **Country of origin** |
| --- | --- | --- | --- |
| DMEM | NA | Biochrom | Berlin, Germany |
| 50x B27 supplement | 0.5x | Thermo Fisher Scientific | Schwerte, Germany |
| Penicillin/streptomycin | 1x | Santa Cruz Biotechnology/ Sigma-Aldrich | Dallas, Texas, USA/Munich, Germany |
| 200 mM glutamine | 2 mM | Sigma-Aldrich | Munich, Germany |
| Vitamin C | 50 µg/ml | Sigma-Aldrich | Munich, Germany |
| FGF10 | 50 ng/ml | Stem Cell Technologies | Cologne, Germany |
| Sant-1 | 0.25 µM | Selleck Chemicals | Munich, Germany |
| All-trans retinoic acid (ATRA) | 2 µM | Sigma-Aldrich | Munich, Germany |
| LDN193189 | 0.5 µM | Sigma-Aldrich | Munich, Germany |

| **Stage 4 medium (144 h, day 9-15)** | **Final concentration** | **Supplier** | **Country of origin** |
| --- | --- | --- | --- |
| DMEM | NA | Biochrom | Berlin, Germany |
| 50x B27 supplement | 0.5x | Thermo Fisher Scientific | Schwerte, Germany |
| Penicillin/streptomycin | 1x | Santa Cruz Biotechnology/ Sigma-Aldrich | Dallas, Texas, USA/Munich, Germany |
| 200 mM glutamine | 2 mM | Sigma-Aldrich | Munich, Germany |
| EGF | 200 ng/ml | Stem Cell Technologies | Cologne, Germany |
| Nicotinamide | 10 mM | Sigma-Aldrich | Munich, Germany |
| LDN193189 | 0.5 µM | Sigma-Aldrich | Munich, Germany |
| Vitamin C | 50 µg/ml | Sigma-Aldrich | Munich, Germany |

| **Stage 5 medium (72 h, day 15-18)** | **Final concentration** | **Supplier** | **Country of origin** |
| --- | --- | --- | --- |
| BE5 stock | NA |  |  |
| ITS-X | 0.5x | Thermo Fisher Scientific | Schwerte, Germany |
| Heparin | 10 µg/ml | Sigma-Aldrich | Munich, Germany |
| Betacellulin | 20 ng/ml | Stem Cell Technologies | Cologne, Germany |
| ZnSO4 | 10 µM | MerckMillipore | Schwalbach, Germany |
| Sant-1 | 0.25 µM | Selleck Chemicals | Munich, Germany |
| All-trans retinoic acid (ATRA) | 50 nM | Sigma-Aldrich | Munich, Germany |
| XXI | 1 µM | Stem Cell Technologies | Cologne, Germany |
| Alk5iII | 10 µM | Santa Cruz Biotechnology | Dallas, Texas, USA |
| GC1 | 1µM | Tocris | Bristol, United Kingdom |
| Staurosporine | 3 nM | Cayman Chemical | Ann Arbor, Michigan, USA |
| ROCK inhibitor Y-27632 | 5 µM | Selleck Chemicals | Munich, Germany |
| LDN193189 | 100 nM | Sigma-Aldrich | Munich, Germany |

| **Stage 6 medium (96 h, day 18-22)** | **Final concentration** | **Supplier** | **Country of origin** |
| --- | --- | --- | --- |
| BE5 stock | NA |  |  |
| ITS-X | 0.5x | Thermo Fisher Scientific | Schwerte, Germany |
| Heparin | 10 µg/ml | Sigma-Aldrich | Munich, Germany |
| Betacellulin | 20 ng/ml | Stem Cell Technologies | Cologne, Germany |
| ZnSO4 | 10 µM | MerckMillipore | Schwalbach, Germany |
| XXI | 1 µM | Stem Cell Technologies | Cologne, Germany |
| Alk5iII | 10 µM | Santa Cruz Biotechnology | Dallas, Texas, USA |
| GC1 | 1 µM | Tocris | Bristol, United Kingdom |
| Staurosporine | 3 nM | Cayman Chemical | Ann Arbor, Michigan, USA |
| ROCK inhibitor Y-27632 | 5 µM | Selleck Chemicals | Munich, Germany |
| LDN193189 | 100 nM | Sigma-Aldrich | Munich, Germany |

| **Stage 7 medium (168 h, day 22-29)** | **Final concentration** | **Supplier** | **Country of origin** |
| --- | --- | --- | --- |
| CMRL-stock | NA |  |  |
| Human insulin | 20 nM | Sigma-Aldrich | Munich, Germany |
| ZnSO4 | 10 µM | MerckMillipore | Schwalbach, Germany |
| Heparin | 10 µg/ml | Sigma-Aldrich | Munich, Germany |
| Ethanolamine | 15 µM | Sigma-Aldrich | Munich, Germany |
| Medium trace elements A | 1:1000 | Corning | Amsterdam, Netherlands |
| Medium trace elements B | 1:1000 | Corning | Amsterdam, Netherlands |
| Chemically defined lipid concentrate | 1:2000 | Thermo Fisher Scientific | Schwerte, Germany |
| Trolox | 10 µM | Sigma-Aldrich | Munich, Germany |
| Alk5iII | 10 µM | Santa Cruz Biotechnology | Dallas, Texas, USA |
| GC1 | 1 µM | Tocris | Bristol, United Kingdom |
| Human apo-transferrin | 70 nM | Sigma-Aldrich | Munich, Germany |

|  |  |  | **Supplier** | **Country of origin** |
| --- | --- | --- | --- | --- |
|  | CMRL-1066 | | Thermo Fisher Scientific | Schwerte, Germany |
| **CMRL-stock** | 2% FAF-BSA | | Serva | Heidelberg, Germany |
|  | 1x penicillin/streptomycin | | Santa Cruz Biotechnology/ Sigma-Aldrich | Dallas, Texas, USA/Munich, Germany |
|  | 2 mM glutamine | | Sigma-Aldrich | Munich, Germany |
|  | 5 mM So-pyruvate | | Capricorn Scientific | Ebsdorfergrund, Germany |

|  |  |  | **Supplier** | **Country of origin** |
| --- | --- | --- | --- | --- |
|  | MCDB131 | | Thermo Fisher Scientific | Schwerte, Germany |
| **BE5-stock** | 2% FAF-BSA | | Serva | Heidelberg, Germany |
|  | 1x penicillin/streptomycin | | Santa Cruz Biotechnology/ Sigma-Aldrich | Dallas, Texas, USA/Munich, Germany |
|  | 2mM glutamine | | Sigma-Aldrich | Munich, Germany |
|  | 20 mM D-(+)-glucose | | Sigma-Aldrich | Munich, Germany |
|  | 1.754g/l NaHCO₃ | | J.T. Baker | Munich, Germany |

**3D production protocol**

| **Stage 1A medium (24 h, day 0-1)** | **Final concentration** | **Supplier** | **Country of origin** |
| --- | --- | --- | --- |
| RPMI 1640 medium | NA | Biochrom | Berlin, Germany |
| 50x B27 supplement | 0.5x | Thermo Fisher Scientific | Schwerte, Germany |
| Penicillin/streptomycin | 1x | Santa Cruz Biotechnology/ Sigma-Aldrich | Dallas, Texas, USA/Munich, Germany |
| 200 mM glutamine | 2 mM | Sigma-Aldrich | Munich, Germany |
| 100x non-essential amino acids | 1x | Thermo Fisher Scientific | Schwerte, Germany |
| 100 mM So-pyruvate | 1 mM | Capricorn Scientific | Ebsdorfergrund, Germany |
| 100x ITS-X | 0.5x | Thermo Fisher Scientific | Schwerte, Germany |
| Vitamin C | 0.25 mM | Sigma-Aldrich | Munich, Germany |
| Activin A | 30 ng/ml | Stem Cell Technologies | Cologne, Germany |
| CHIR99021 | 3 µM | Cayman Chemical | Ann Arbor, Michigan, USA |

| **Stage 1B medium (72 h, day 1-4)** | **Final concentration** | **Supplier** | **Country of origin** |
| --- | --- | --- | --- |
| RPMI 1640 medium | NA | Biochrom | Berlin, Germany |
| 50x B27 supplement | 0.5x | Thermo Fisher Scientific | Schwerte, Germany |
| Penicillin/streptomycin | 1x | Santa Cruz Biotechnology/ Sigma-Aldrich | Dallas, Texas, USA/Munich, Germany |
| 200 mM glutamine | 2 mM | Sigma-Aldrich | Munich, Germany |
| 100x non-essential amino acids | 1x | Thermo Fisher Scientific | Schwerte, Germany |
| 100 mM So-pyruvate | 1 mM | Capricorn Scientific | Ebsdorfergrund, Germany |
| 100x ITS-X | 0.5x | Thermo Fisher Scientific | Schwerte, Germany |
| Vitamin C | 0.25 mM | Sigma-Aldrich | Munich, Germany |
| Activin A | 30 ng/ml | Stem Cell Technologies | Cologne, Germany |

| **Stage 2 medium (96 h, day 4-8)** | **Final concentration** | **Supplier** | **Country of origin** |
| --- | --- | --- | --- |
| Advanced RPMI 1640 medium | NA | Thermo Fisher Scientific | Schwerte, Germany |
| 50x B27 supplement | 0.5x | Thermo Fisher Scientific | Schwerte, Germany |
| Penicillin/streptomycin | 1x | Santa Cruz Biotechnology/ Sigma-Aldrich | Dallas, Texas, USA/Munich, Germany |
| 200 mM glutamine | 2 mM | Sigma-Aldrich | Munich, Germany |
| Vitamin C | 0.25 mM | Sigma-Aldrich | Munich, Germany |
| FGF7 | 5 ng/ml | Stem Cell Technologies | Cologne, Germany |
| IWR-1 | 2 µM | Selleck Chemicals | Munich, Germany |
| LDN193189 | 0.5 µM | Sigma-Aldrich | Munich, Germany |
| All-trans retinoic acid (ATRA) | 1 µM | Sigma-Aldrich | Munich, Germany |

| **Stage 3 medium (24 h, day 8-9)** | **Final concentration** | **Supplier** | **Country of origin** |
| --- | --- | --- | --- |
| DMEM | NA | Biochrom | Berlin, Germany |
| 50x B27 supplement | 0.5x | Thermo Fisher Scientific | Schwerte, Germany |
| Penicillin/streptomycin | 1x | Santa Cruz Biotechnology/ Sigma-Aldrich | Dallas, Texas, USA/Munich, Germany |
| 200 mM glutamine | 2 mM | Sigma-Aldrich | Munich, Germany |
| IWR-1 | 2 µM | Selleck Chemicals | Munich, Germany |
| FGF10 | 50 ng/ml | Stem Cell Technologies | Cologne, Germany |
| Sant-1 | 0.25 µM | Selleck Chemicals | Munich, Germany |
| All-trans retinoic acid (ATRA) | 2 µM | Sigma-Aldrich | Munich, Germany |
| LDN193189 | 0.5 µM | Sigma-Aldrich | Munich, Germany |
| Vitamin C | 50 µg/ml | Sigma-Aldrich | Munich, Germany |

| **Stage 4 medium (144 h, day 9-15)** | **Final concentration** | **Supplier** | **Country of origin** |
| --- | --- | --- | --- |
| DMEM | NA | Biochrom | Berlin, Germany |
| 50x B27 supplement | 0.5x | Thermo Fisher Scientific | Schwerte, Germany |
| Penicillin/streptomycin | 1x | Santa Cruz Biotechnology/ Sigma-Aldrich | Dallas, Texas, USA/Munich, Germany |
| 200 mM glutamine | 2 mM | Sigma-Aldrich | Munich, Germany |
| IWR-1 | 2 µM | Selleck Chemicals | Munich, Germany |
| EGF | 100 ng/ml | Stem Cell Technologies | Cologne, Germany |
| Nicotinamide | 10 mM | Sigma-Aldrich | Munich, Germany |
| LDN193189 | 0.5 µM | Sigma-Aldrich | Munich, Germany |
| Vitamin C | 50 µg/ml | Sigma-Aldrich | Munich, Germany |

*Analysis of GFP2 expression on day 12 and transfer from 2D into 3D orbital shaking culture

Stage 5, 6 and 7 without changes from the 2D experimental protocol.
