# Supplementary material for: New hPSC SOX9 and INS Reporter Cell Lines Facilitate the Observation and Optimization of Differentiation into Insulin-Producing Cells
Source: Stem Cell Rev Rep. 2021 Aug 19;17(6):2193–209. doi: 10.1007/s12015-021-10232-9 (PMC8599335; doi:10.1007/s12015-021-10232-9)
Supplement: Supplementary file 2 — Supplementary file2 (DOCX 3.56 mb) [file 12015_2021_10232_MOESM2_ESM.docx]

**Supplementary data**

**
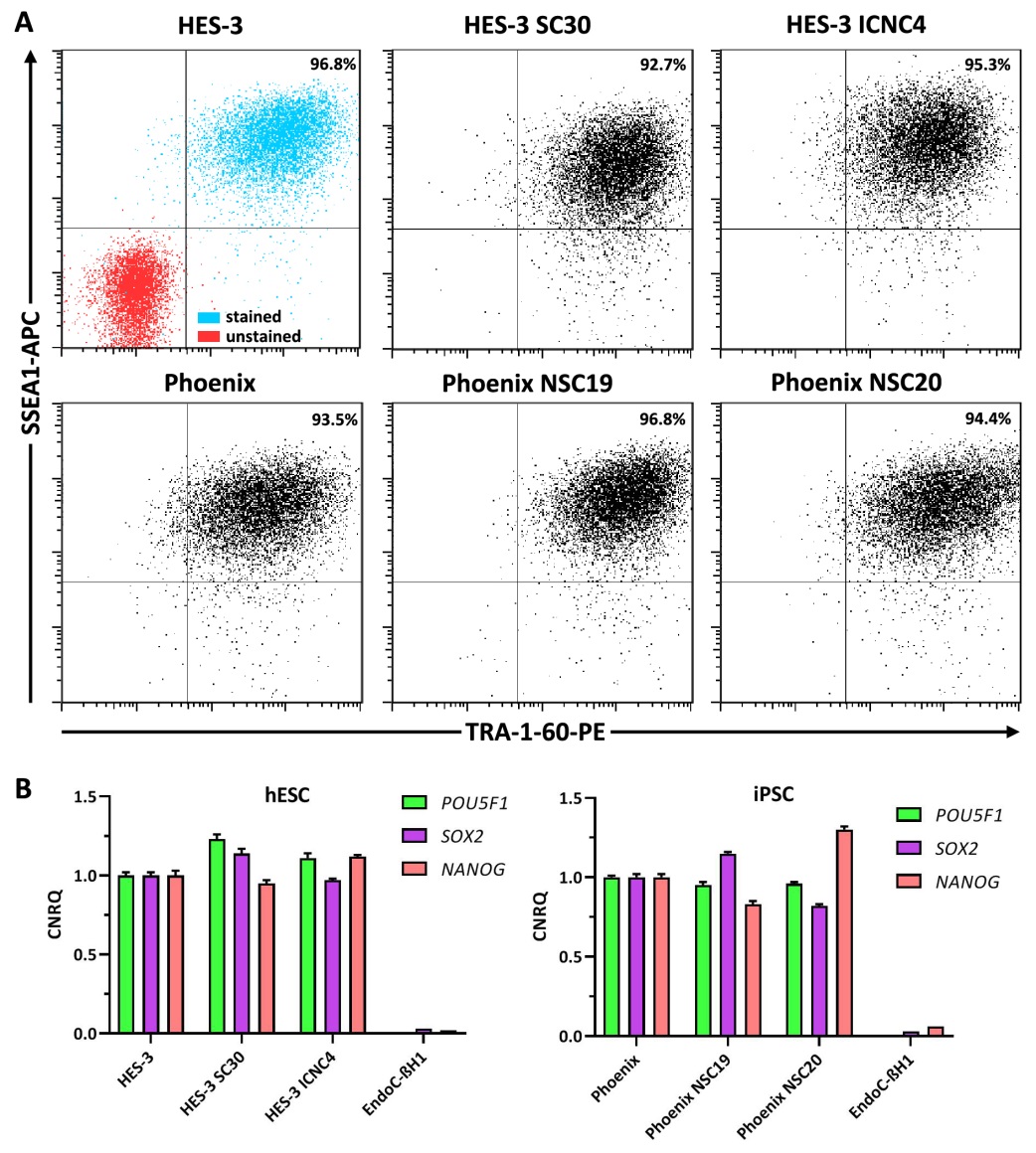
**

**Supplementary figure 1. Characterization of the generated clonal cell lines.** (**A**) Flow cytometric quantification of SSEA1-APC (allophycocyanin) and TRA-1-60-PE (phycoerythrin) in hESC (HES-3) and iPSC (Phoenix) and the cell clones derived therefrom. (**B**) Relative gene expression of *POU5F1* (Oct4), *SOX2*, and *NANOG* in hESC and iPSC and the cell clones derived therefrom compared to the non-pluripotent insulin-producing cell line EndoC-βH1. Data are means ± SEM from a triplicate measurement.

**
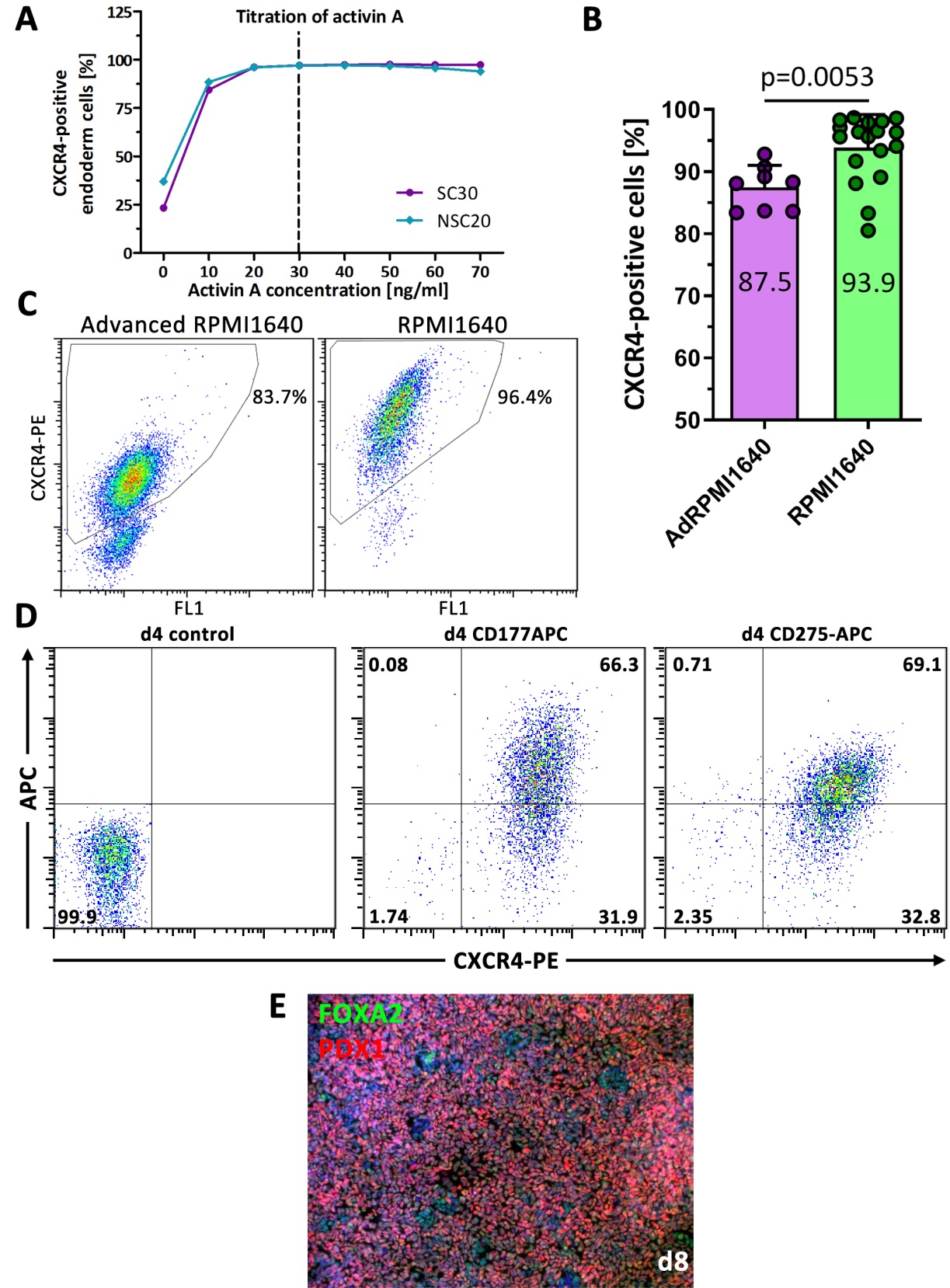
**

**Supplementary figure 2. Endoderm and pancreatic-duodenal differentiation efficiency.** (**A**) Titration of the optimal activin A concentration for differentiation of hPSC into CXCR4-positive endoderm cells. (**B**) Flow cytometric quantification of CXCR4 at d4 of endoderm differentiation. Data are means ± SEM, n= 8-18, two-tailed *Student's* t-test, ** p < 0.01. Differentiation protocol based on [[9](#_ENREF_9)]. (**C**) Representative flow cytometry dot plots of CXCR4 staining in two endoderm differentiation media. (**D**) Double flow cytometric staining of CD177-APC/CXCR4-PE and CD275-APC/CXCR4-PE at d4 of endoderm differentiation. (**E**) PDX1/FOXA2 double-positive pancreatic duodenal cells at d8 of differentiation. Differentiation protocol based on [[7](#_ENREF_7)] and [[8](#_ENREF_8)].

**
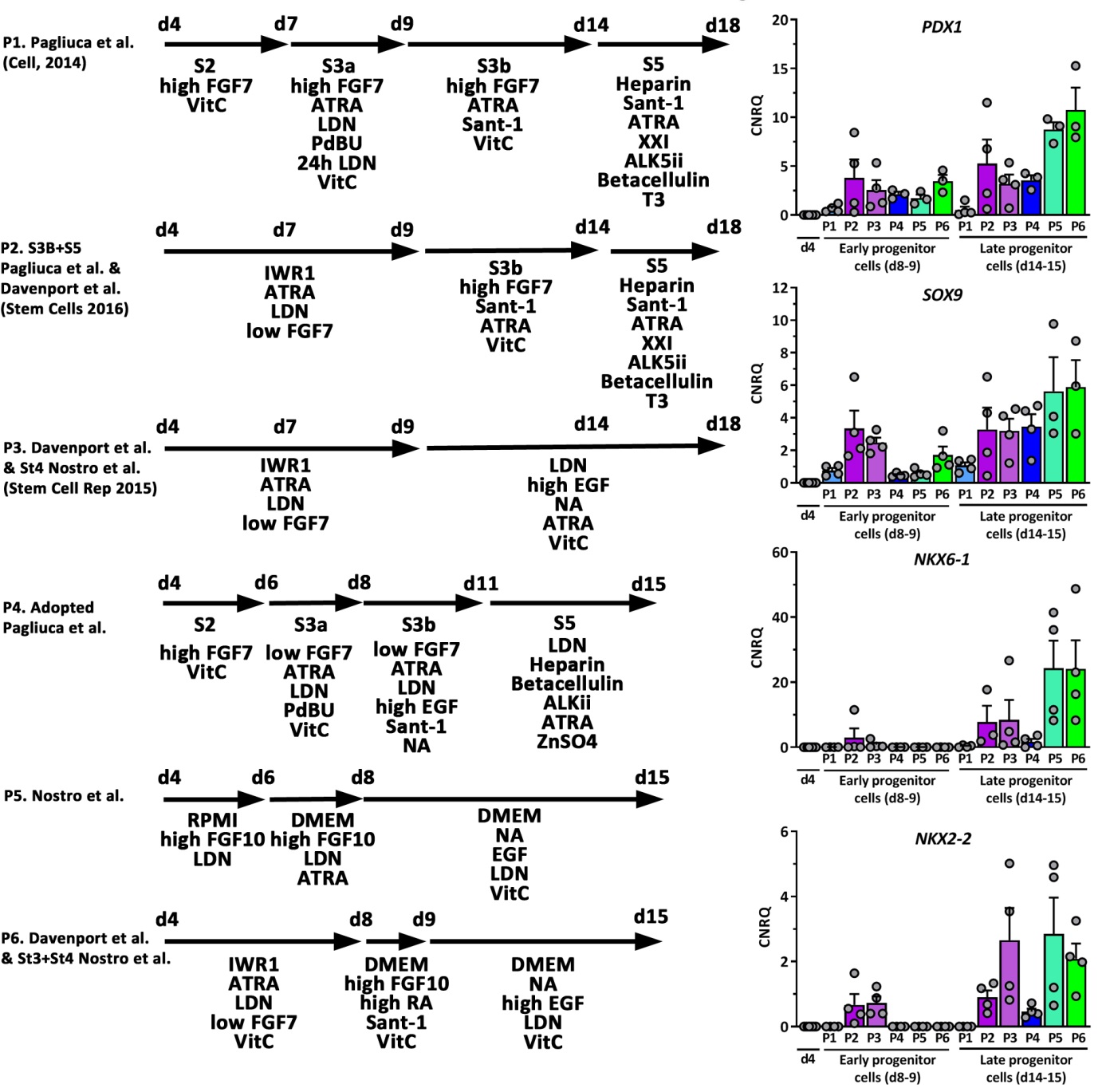
**

**Supplementary figure 3. Comparison of six adopted differentiation protocols for the generation of MPCs from hPSC.**

Depicted is the relative gene expression of *PDX1*, *SOX9*, *NKX6-1*, and *NKX2-2*. Data are means ± SEM. n= 3-4. The differentiation protocols were adopted from [[7](#_ENREF_7)], [[8](#_ENREF_8)], [[11](#_ENREF_11)] and [[14](#_ENREF_14)].

**
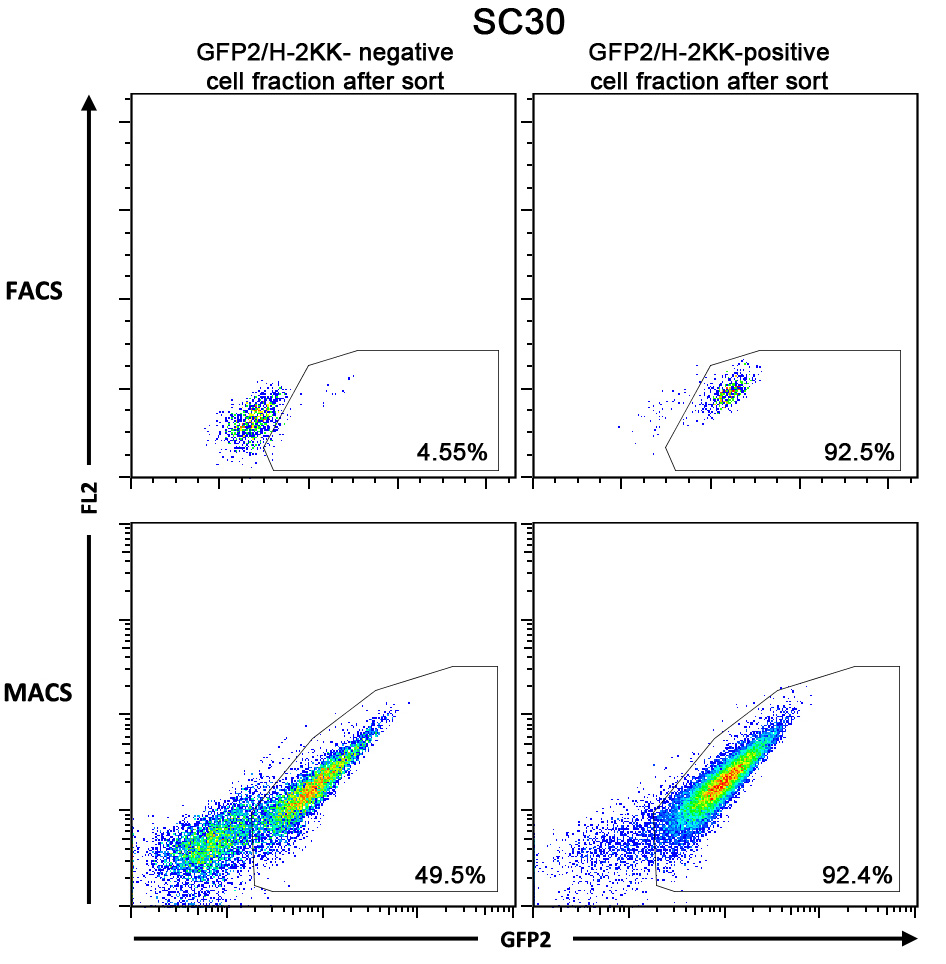
**

**Supplementary figure 4. Representative dot plot presentation of cell sorting experiments using the SC30 cell clone.** GFP2^+^ pancreatic progenitors can be sorted by FACS (upper images) or MACS (upper images) with comparable efficiencies.

**
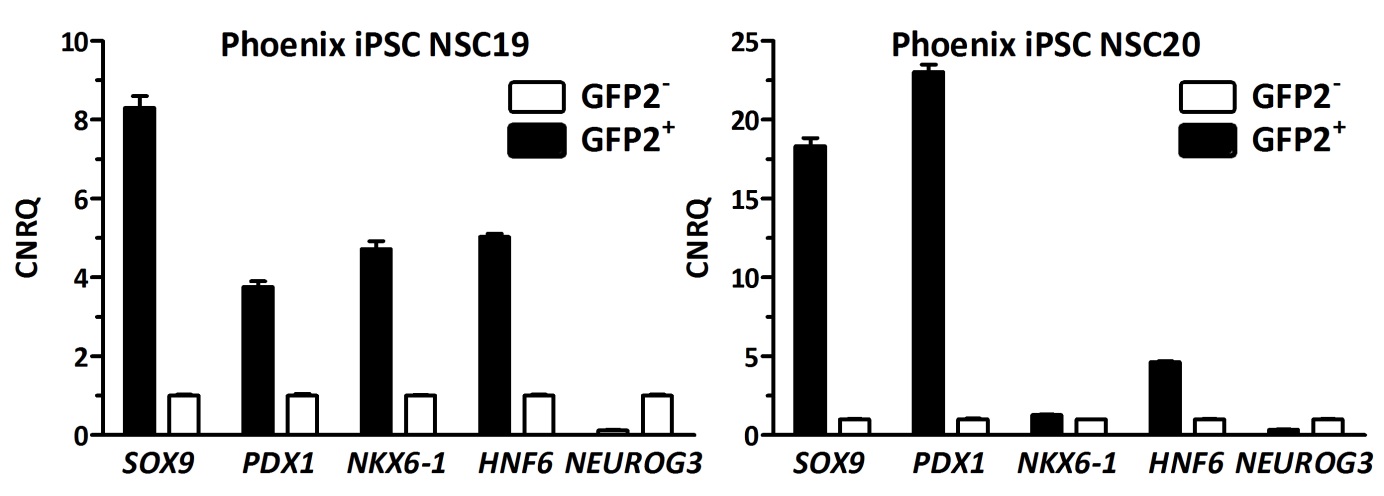
**

**Supplementary figure 5. RT-qPCR analysis of sorted NSC19 and NSC20 derived** **GFP2^+^ and GFP2^-^ cells at d12 of differentiation.** Depicted is the relative gene expression of *SOX9*, *PDX1*, *NKX6-1*, *HNF6*, and *NEUROG3*. Data are means ± SD from a single sorting experiment measured in triplicate.

**
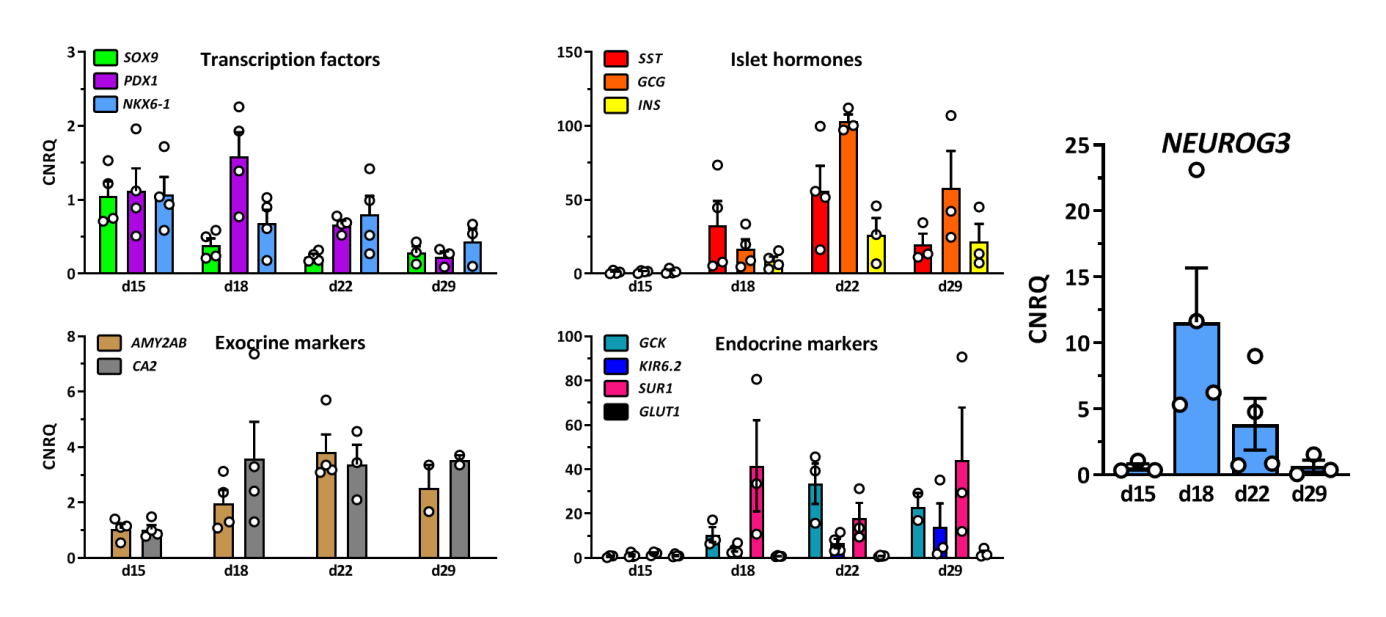
**

**Supplementary figure 6. RT-qPCR analysis of sorted GFP2^+^ cells after MACS at day 12 of differentiation using the SC30 cell clone.** Further differentiation was conducted according to the 2D experimental protocol. Depicted is the relative gene expression of the transcription factors*, SOX9, PDX1, NKX6-1* and *NEUROG3,* the islet hormones somatostatin (*SST*), glucagon (*GCG*) and insulin (*INS*), the exocrine marker genes amylase 2 (*AMY2AB*) and carbonic anhydrase 2 (*CA2*) and the endocrine marker genes glucokinase (*GCK*), *KIR6.2*, *SUR1* and *GLUT2*. Data are means ± SEM, n= 2-4. Data are normalized to housekeeping genes and d15 samples scaled to 1.

**
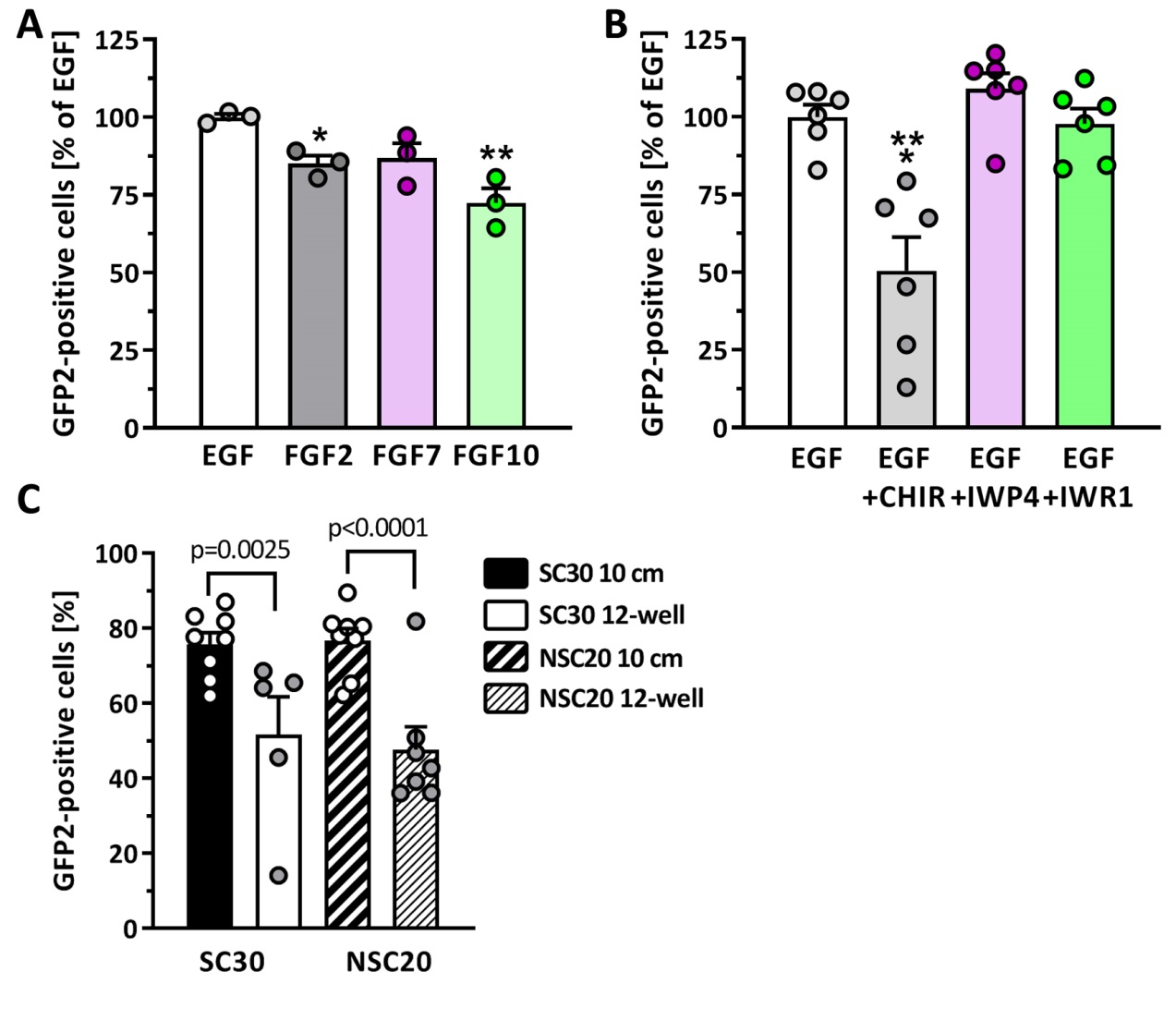
**

**Supplementary figure 7. Effect of growth factors, Wnt/beta-catenin signaling and upscaling on the generation of GFP2^+^ pancreatic progenitors.**

(**A**) Effect of different growth factors each used at 100 ng/ml on GFP2 expression in NSC20 cells. Data are means ± SEM. n= 3, ANOVA plus *Dunnett’s* post-hoc test, ** p < 0.01, * p < 0.05. (**B**) Effect of canonical Wnt-signaling on GFP2 expression in NSC20 cells. The pathway was activated by CHIR (3 µM) or inhibited by IWP4 (1 µM) or IWR-1 (2 µM). Data are means ± SEM, n= 6, ANOVA plus *Dunnett’s* post-hoc test, *** p < 0.01 (**C**) Flow cytometric quantification of GFP2^+^ pancreatic progenitors from the cell lines SC30 and NSC20 at d12 of differentiation differentiated in 12-well plate cavities or 10 cm cell culture dishes. Data are means ± SEM, n= 8-11. Two-tailed *Student’s* t-test.

**
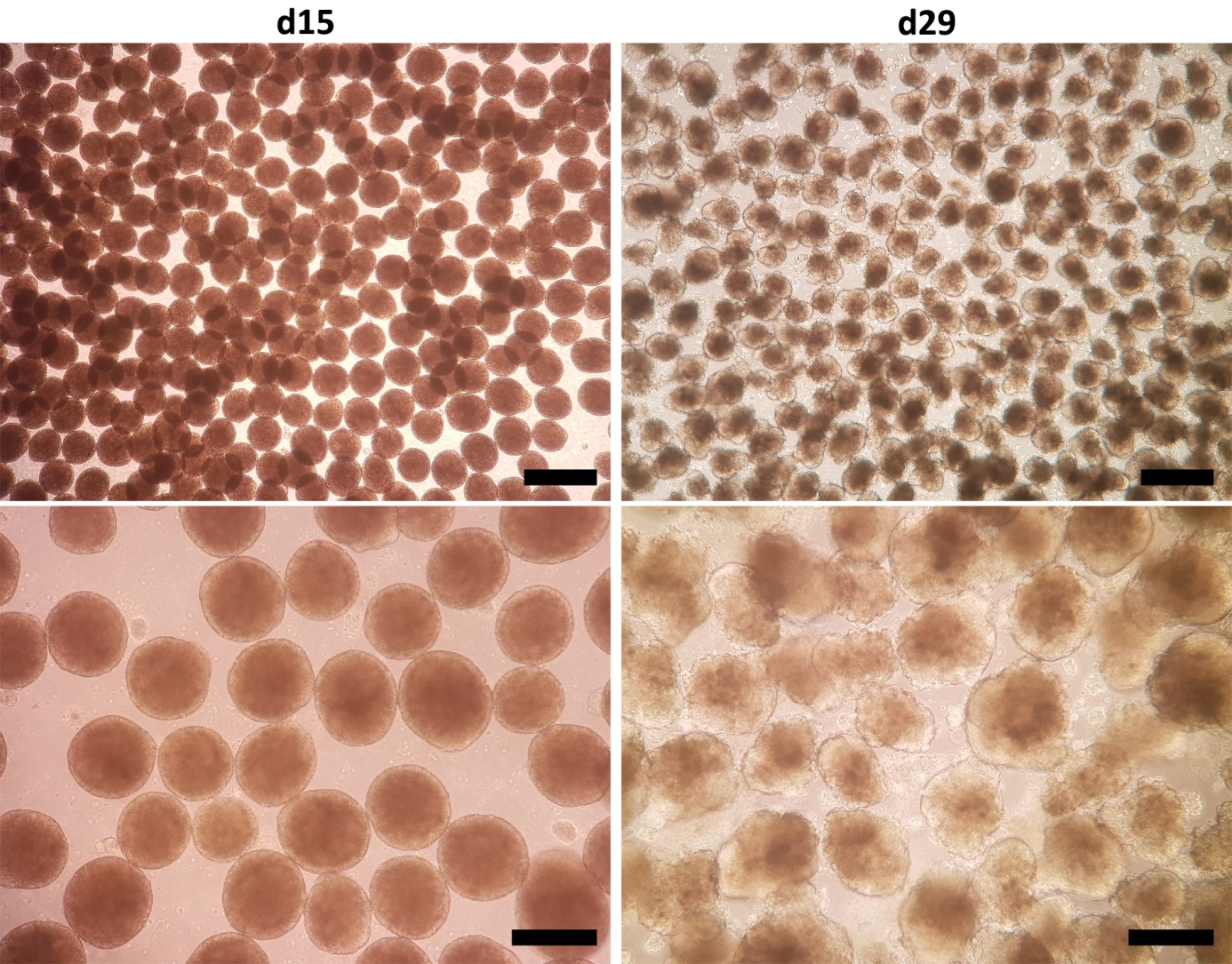
**

**Supplementary figure 8. Generation of pancreatic spheroids (left images) and stem cell-derived organoids (right images) by 3D shaking culture.** Shown are representative images of cell spheroids 3 days (left) and organoids 17 days (right) after transfer from 2D adherent culture to 3D orbital suspension culture at 100 rpm. Scale bar for lower magnification image = 500 µm. Scale bar for higher magnification image = 200 µm.

**
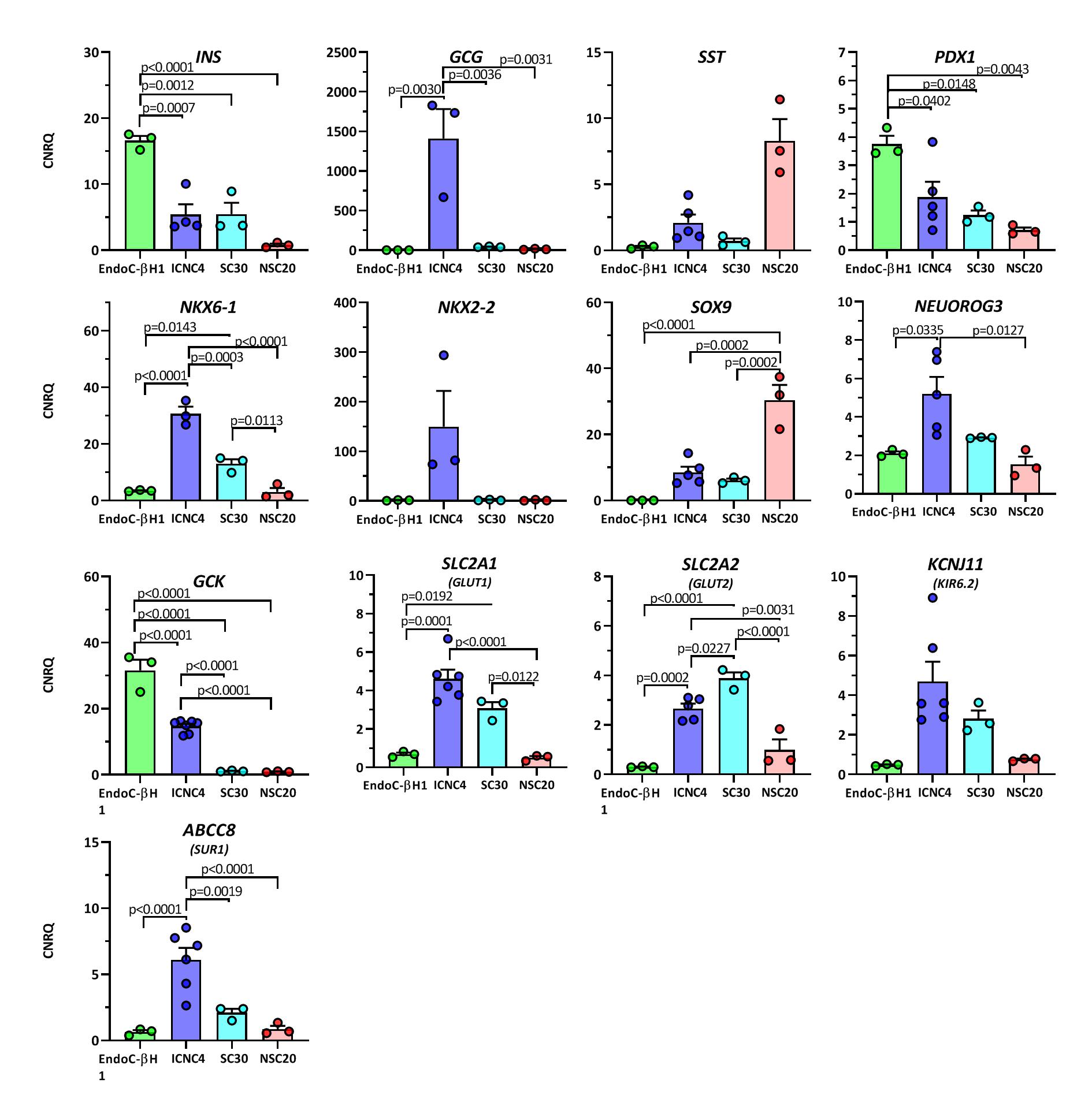
**

**Supplementary figure 9. Relative gene expression of pancreatic and endocrine genes in NSC20-, ICNC4- and SC30-derived organoids after 3D differentiation compared to EndoC-βH1 cells**. Data are means ± SEM. ANOVA one-way analysis of variance.

**
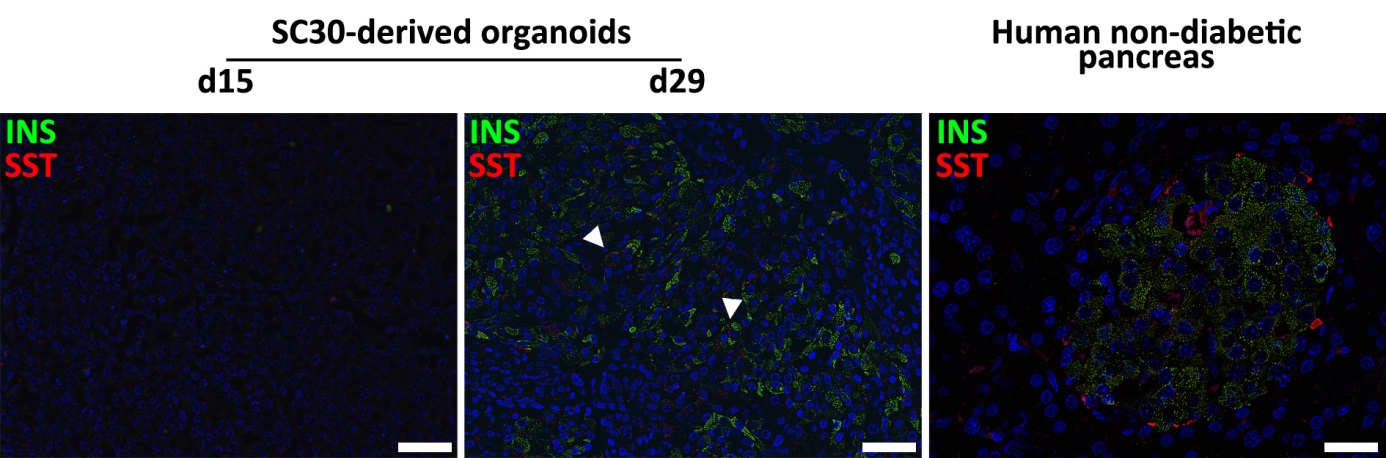
**

**Supplementary figure 10. Immunhistochemical analysis of SC-derived pancreatic organoids.** D15 spheroids and d29 stem cell-derived organoids derived in 3D from the SC30 clone were fixed, sectioned and double-stained for somatostatin (red) and insulin (green). A human non-diabetic pancreas was taken as control. Arrowheads mark polyhormonal cells. Scale Bar = 50 µm.

**
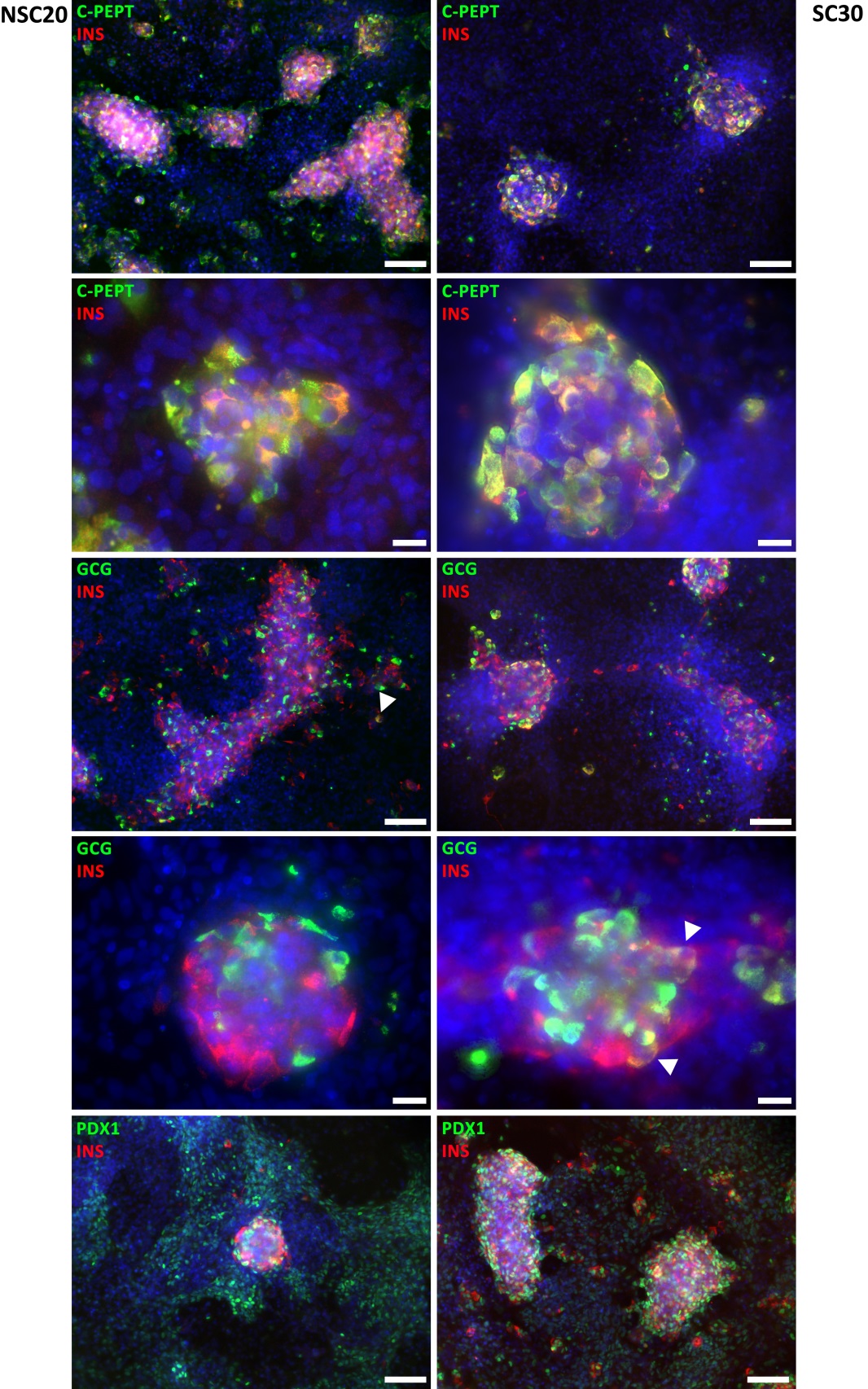
**

**Supplementary figure 11.** **Immunofluorescence staining of NSC20-derived (left images) and SC30-derived (right images) pancreatic and endocrine cells at d29 using the production protocol in 2D.** Double staining of insulin (red) and C-peptide, glucagon and PDX1 (all in green). Counterstaining with DAPI. Scale bar for lower magnification image = 100 µM, scale bar for higher magnification images = 20 µM. Arrowheads indicate polyhormonal insulin and glucagon co-expressing cells.

**
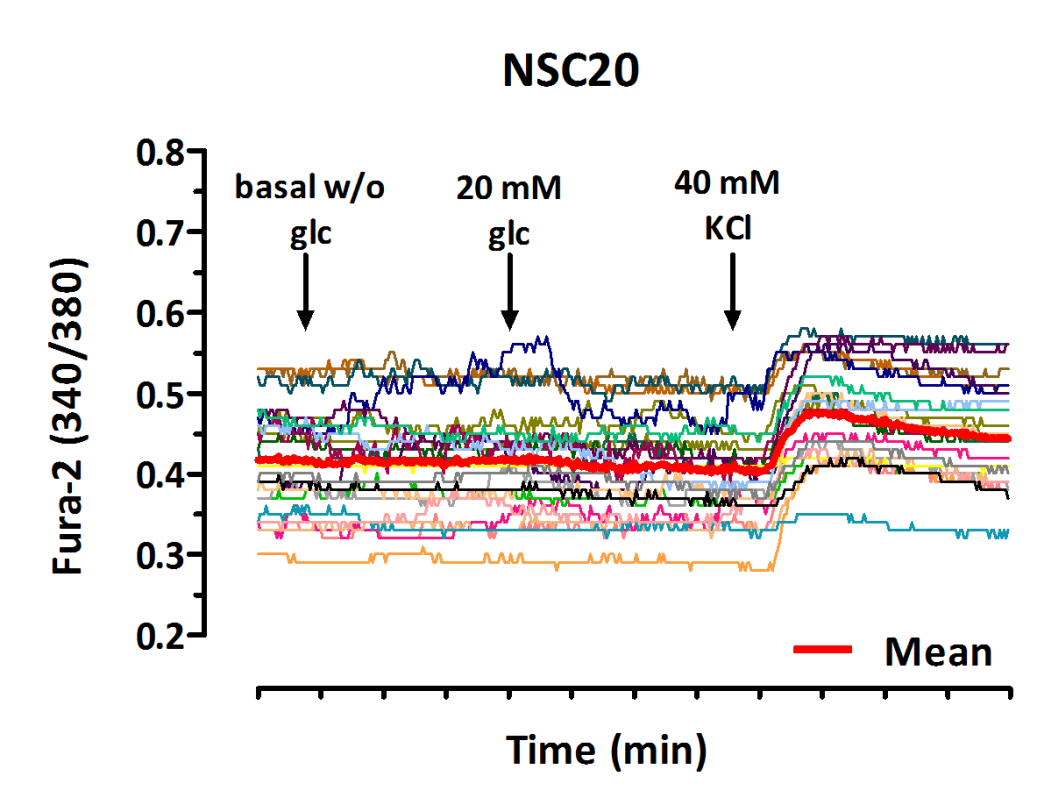
**

**Supplementary figure 12.** **Recording of the Fura-2/AM emission ratio at 340 and 380** **nm over 13 minutes.** NSC20-derived organoids were dissociated, seeded on glass slides for 24 h and loaded with Fura-2/AM. Then the cells were stimulated with basal KR ∅ glucose, 20 mM glucose in KR, basal KR ∅ glucose and finally KR plus 40 mM KCl. Mean value of all 15 cells in bold red.

**Supplementary Table 1:** sgRNAs for HDR

| **Gene Symbol/ Cas type** | **Orientation** | **Primer Sequence 5’-3’** |
| --- | --- | --- |
| *SOX9*  Cas9  T5 | Forward cloning primer | CACCG**ACACAGCTCACTCGACCTTGAGG** |
|  | Reverse cloning primer | AAACCAAGGTCGAGTGAGCTGTGTC |
| *INS*  Cas9n  #1  Nickase pair 1 | Forward cloning primer | CACCG**TGCAACTAGACGCAGCCCGC** |
|  | Reverse cloning primer | AAACGCGGGCTGCGTCTAGTTGCAC |
| *INS*  Cas9n  #23  Nickase pair 1 | Forward cloning primer | CACCG**CTGGTAGAGGGAGCAGATGC** |
|  | Reverse cloning primer | AAACGCATCTGCTCCCTCTACCAGC |
| *INS*  Cas9n  #10  Nickase pair 2 | Forward cloning primer | CACCG**CCTCCTGCACCGAGAGAGA** |
|  | Reverse cloning primer | AAACTCTCTCTCTGGTGCAGGAGGC |
| *INS*  Cas9n  #7  Nickase pair 2 | Forward cloning primer | CACCG**AGTTGCAGTAGTTCTCCAGC** |
|  | Reverse cloning primer | AAACGCTGGAGAACTACTGCAACTC |

sgRNA sequences marked in bold

**Supplementary Table 2:** Primer pairs for gene expression analysis.

| **Gene** | **Primer Sequence 5’-3’** | **Accession #** |
| --- | --- | --- |
| *ABCC8*  *(SUR1)* | Fw: tcacaccgctgttcctgct  Rev: agaaggagcgaggacttgcc | NM_001287174.2 |
| *CPA1* | Fw: caggctccctctgtattggc  Rev: ggacttgacctccacttcgg | NM_001868.4 |
| *G6PD* | Fw: aggccgtcaccaagaacattca Rev: cgatgatgcggttccagcctat | NM_000402 |
| *GCG* | Fw: aagcatttactttgtggctggatt  Rev: tgatctggatttctcctctgtgtct | NM_002054.5 |
| *GCK* | Fw: cctgggtggcactaacttcag  Rev: tagtcgaagagcatctcagca | NM_000162.5 |
| *INS* | Fw: gccccgagatacatcagagg  Rev: ccaggtcacccaggactttac | NM_000207.3 |
| *KCNJ11*  *(KIR6.2)* | Fw: aaggaagagtctggtgggga  Rev: tagggcctcactgcagagtc | NM_000525.4 |
| *NANOG* | Fw: aatacctcagcctccagcagatg Rev: tgcgtcacaccattgctattcttc | NM_024865 |
| *NEUROG3* | Fw: gcgaccagaagcccgctg  Rev: ggcgtcatcctttctaccggc | NM_020999.4 |
| *NKX2-2* | Fw: aaccccttctacgacagcagcg  Rev: acttggagcttgagtcctgagggg | NM_002509 |
| *NKX6-1* | Fw: ggcccggagtgatgcagagc  Rev: tcttcccgtctttgtccaac | NM_006168.3 |
| *ONECUT1*  *(HNF6)* | Fw: cgctccgcttagcagcatgc Rev: gtgtgttgcctctatccttcccatg | NM_004498 |
| *PDX1* | Fw: cgttccagctgcctttcccat  Rev: ccgtgagatgtacttgttgaatagga | NM_000209 |
| *POU5F1* | Fw: cttgctgcagaagtgggtggagg Rev: ctgcagtgtgggtttcgggca | NM_002701 |
| *SLC2A1*  *(GLUT1)* | Fw: cctgcagtttggctacaaca  Rev: aggatgctctccccatagcg | NM_006516 |
| *SLC2A2*  *(GLUT2)* | Fw: actgggaccctggttttca  Rev: ccagtggaacacccaaaaca | NM_000340 |
| *SOX2* | Fw: agctacagcatgatgcagga Rev: ggtcatggagttgtactgca | NM_003106 |
| *SOX9* | Fw: gcggaggaagtcggtgaagaacg  Rev: ctgggattgccccgagtgctc | NM_000346 |
| *SST* | Fw: cccagactccgtcagtttc  Rev: tccgtctggttgggttag | NM_001048.4 |
| *TBP* | Fw: caacagcctgccaccttacgctc Rev: aggctgtggggtcagtccagtg | NM_003194 |
| *TUBA1A* | Fw: ggcagtgtttgtagacttggaaccc Rev: tgtgataagttgctcagggtggaag | NM_006009 |

**Supplementary Table 3:** Primary and conjugated antibodies used in this study.

| **Protein** | **Supplier** | **Cat #** | **Dilution** |
| --- | --- | --- | --- |
| CPA1 | Origene | TA500053 | 1:100 |
| CK19 | R&D systems | AF3506 | 1:300 |
| C-peptide | Thermo Fisher | MA1-19159 | 1:400 |
| Glucagon | Abcam | ab10988 | 1:300 – 1:2000 |
| Insulin | Agilent DAKO  Abcam | A564  ab7842 | 1:100  1:100 |
| NEUROG3 | R&D systems | AF3444 | 1:200 |
| NKX6-1 | R&D systems | AF5857 | 1:100 – 1:300 |
| ONECUT1  (HNF6) | R&D systems | AF6277 | 1:100 |
| PDX1 | R&D systems | AF2419 | 1:300 |
| Somatostatin | Abcam | ab22682 | 1:500 - 1:1000 |
| SOX9 | R&D systems | AF3075 | 1:300 |
| Anti-SSEA4-APC | Miltenyi Biotec | 130-098-347 | * |
| Anti-Tra-1-60-PE | Miltenyi Biotec | 130-122-965 | * |
| anti CXCR4-PE | Neuromics | FC15004 | * |
| anti CXCR4-APC | Miltenyi Biotec | 130-098-357 | * |
| Anti-CD177-APC | Miltenyi Biotec | 130-101-512 | * |
| Anti-CD275-APC | Miltenyi Biotec | 130-098-738 | * |
| anti-CD200-APC | Miltenyi Biotec | 130-118-203 | * |
| anti-CD142-PE-Vio616 | Miltenyi Biotec | 130-115-720 | * |
| Anti-GP2 | MBL international | D277-3 | 1:500 |

*according to manufacturer specifications

Supplementary Table 4: Data on the non-diabetic human pancreas organ donors.

|  | **Donor 1** | **Donor 2** | **Donor 3** | **Donor 4** |
| --- | --- | --- | --- | --- |
| Age (years) | 60 | 47 | 64 | 52 |
| Gender | female | male | male | female |
| Cause of pancreas removal | Donor organ | Pancreas resection | Pancreas resection | Pancreas resection |
| Blood glucose conc. (mmol/l) before organ collection | 5.7 | 6.2 | 5.8 | 5.3 |

Pancreases from non-diabetic Caucasian donors (55.8 + 3.8 years; 2 male, 2 female) were obtained in Hannover from an organ donor (#1) in 2007 and from three patients (#2 - #4) between 2009 and 2013 during surgery for organ resection. Tissue was handled and processed according to the recommendations of the Hannover Medical School Ethics Committee.
