# Supplementary material for: New hPSC SOX9 and INS Reporter Cell Lines Facilitate the Observation and Optimization of Differentiation into Insulin-Producing Cells
Source: Stem Cell Rev Rep. 2021 Aug 19;17(6):2193–209. doi: 10.1007/s12015-021-10232-9 (PMC8599335; doi:10.1007/s12015-021-10232-9)

Karyotype Report

**Published 2021-03-18**

| **Sample** | **Sex** | **Status** |
| --- | --- | --- |
| HES-3 | Female | Abnormal |
| NSC20 | Female | Abnormal |
| NSC19 | Female | Abnormal |
| iPSC | Female | Abnormal |
| SC30 | Female | Abnormal |
| ICNC4 | Female | Abnormal |


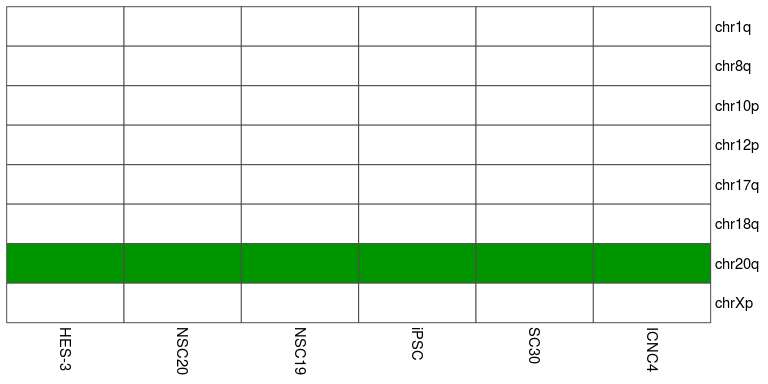


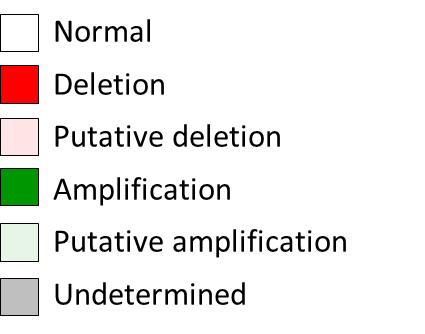


##### .

| **Gene** | **Description** |
| --- | --- |
| **HES-3** | **Chr20q has an amplification in a minimal critical region with a calculated copy number of 3.61.** Amplification of chr20q is a common abnormality seen in hPSC cultures. This routinely involves a minimal amplification at a centrometic region of the q-arm. Note, classical G-band karyotyping often does not have the resolution to accurately detect this abnormality. Whole chromosome duplications have also been observed for chr20. |
| **NSC20** | **Chr20q has an amplification in a minimal critical region with a calculated copy number of 2.74.** Amplification of chr20q is a common abnormality seen in hPSC cultures. This routinely involves a minimal amplification at a centrometic region of the q-arm. Note, classical G-band karyotyping often does not have the resolution to accurately detect this abnormality. Whole chromosome duplications have also been observed for chr20. |
| **NSC19** | **Chr20q has an amplification in a minimal critical region with a calculated copy number of 2.64.** Amplification of chr20q is a common abnormality seen in hPSC cultures. This routinely involves a minimal amplification at a centrometic region of the q-arm. Note, classical G-band karyotyping often does not have the resolution to accurately detect this abnormality. Whole chromosome duplications have also been observed for chr20. |
| **iPSC** | **Chr20q has an amplification in a minimal critical region with a calculated copy number of 2.99.** Amplification of chr20q is a common abnormality seen in hPSC cultures. This routinely involves a minimal amplification at a centrometic region of the q-arm. Note, classical G-band karyotyping often does not have the resolution to accurately detect this abnormality. Whole chromosome duplications have also been observed for chr20. |
| **SC30** | **Chr20q has an amplification in a minimal critical region with a calculated copy number of 4.58.** Amplification of chr20q is a common abnormality seen in hPSC cultures. This routinely involves a minimal amplification at a centrometic region of the q-arm. Note, classical G-band karyotyping often does not have the resolution to accurately detect this abnormality. Whole chromosome duplications have also been observed for chr20. |
| **ICNC4** | **Chr20q has an amplification in a minimal critical region with a calculated copy number of 4.45.** Amplification of chr20q is a common abnormality seen in hPSC cultures. This routinely involves a minimal amplification at a centrometic region of the q-arm. Note, classical G-band karyotyping often does not have the resolution to accurately detect this abnormality. Whole chromosome duplications have also been observed for chr20. |

##### .


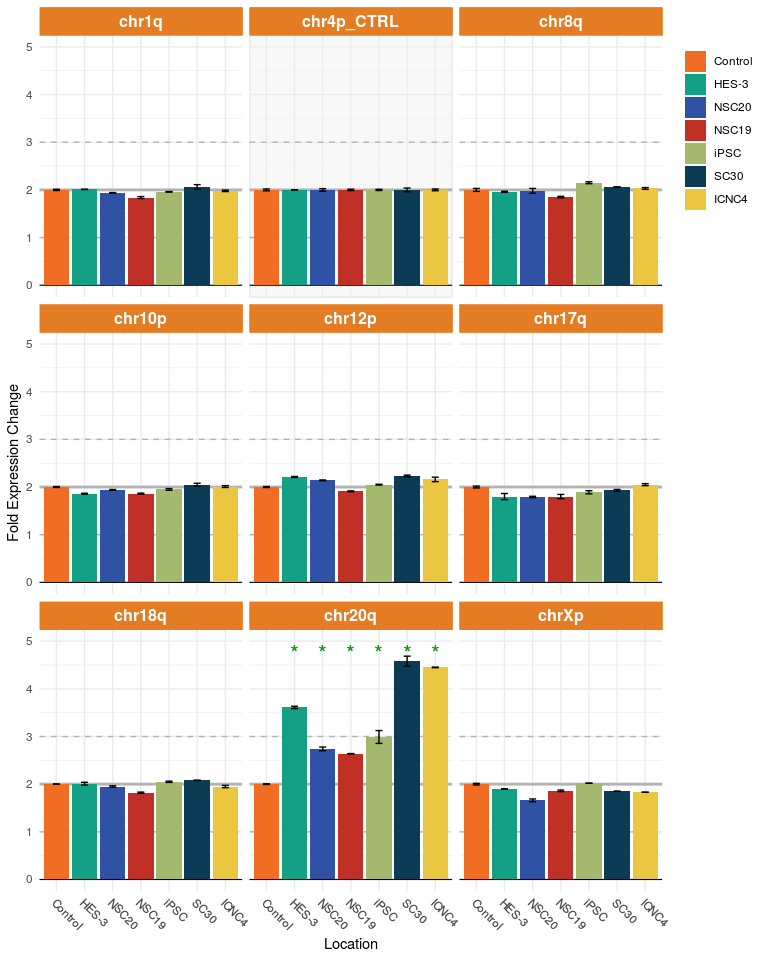

Supplement: Supplementary file 3 — Supplementary file3 (DOCX 105 kb) [file 12015_2021_10232_MOESM3_ESM.docx]
